# Unraveling the evolutionary history of the phosphoryl-transfer chain of the phosphoenolpyruvate:phosphotransferase system through phylogenetic analyses and genome context

**DOI:** 10.1186/1471-2148-8-147

**Published:** 2008-05-16

**Authors:** Iñaki Comas, Fernando González-Candelas, Manuel Zúñiga

**Affiliations:** 1 Instituto Cavanilles de Biodiversidad y Biología Evolutiva, Universidad de Valencia, Valencia, Spain; 2 Instituto de Agroquímica y Tecnología de Alimentos, CSIC, Valencia, Spain

## Abstract

**Background:**

The phosphoenolpyruvate phosphotransferase system (PTS) plays a major role in sugar transport and in the regulation of essential physiological processes in many bacteria. The PTS couples solute transport to its phosphorylation at the expense of phosphoenolpyruvate (PEP) and it consists of general cytoplasmic phosphoryl transfer proteins and specific enzyme II complexes which catalyze the uptake and phosphorylation of solutes. Previous studies have suggested that the evolution of the constituents of the enzyme II complexes has been driven largely by horizontal gene transfer whereas vertical inheritance has been prevalent in the general phosphoryl transfer proteins in some bacterial groups. The aim of this work is to test this hypothesis by studying the evolution of the phosphoryl transfer proteins of the PTS.

**Results:**

We have analyzed the evolutionary history of the PTS phosphoryl transfer chain (PTS-ptc) components in 222 complete genomes by combining phylogenetic methods and analysis of genomic context. Phylogenetic analyses alone were not conclusive for the deepest nodes but when complemented with analyses of genomic context and functional information, the main evolutionary trends of this system could be depicted.

**Conclusion:**

The PTS-ptc evolved in bacteria after the divergence of early lineages such as *Aquificales*, *Thermotogales *and *Thermus/Deinococcus*. The subsequent evolutionary history of the PTS-ptc varied in different bacterial lineages: vertical inheritance and lineage-specific gene losses mainly explain the current situation in *Actinobacteria *and *Firmicutes *whereas horizontal gene transfer (HGT) also played a major role in *Proteobacteria*. Most remarkably, we have identified a HGT event from *Firmicutes *or *Fusobacteria *to the last common ancestor of the *Enterobacteriaceae*, *Pasteurellaceae*, *Shewanellaceae *and *Vibrionaceae*. This transfer led to extensive changes in the metabolic and regulatory networks of these bacteria including the development of a novel carbon catabolite repression system. Hence, this example illustrates that HGT can drive major physiological modifications in bacteria.

## Background

The phosphoenolpyruvate:carbohydrate phosphotransferase system (PTS) was originally described as a sugar phosphorylation system [1] and it represents hitherto the only example of group-translocating transport systems [2]. The PTS couples solute transport to its phosphorylation at the expense of phosphoenolpyruvate (PEP) and it also plays a central role in the regulation of a number of cell processes in some bacteria [3-6]. This system consists of general cytoplasmic energy-coupling proteins, enzyme I (EI) and HPr, and specific enzyme II complexes, which catalyze the uptake and phosphorylation of solutes [3,7]. In turn, enzyme II complexes consist of three functional subunits, IIA, IIB and IIC, although those belonging to the mannose family contain an additional subunit, IID. These complexes have been divided into seven classes on the basis of their amino acid sequence and structural properties [3,7-9].

**Figure 1:**
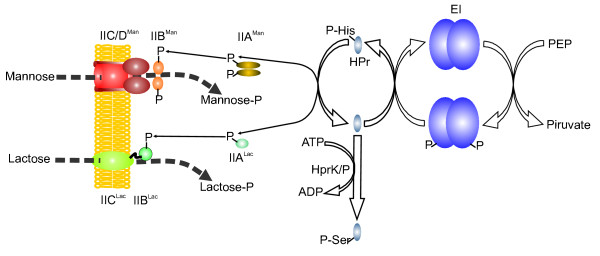
**Schematic representation of the PTS phosphoryl transfer chain**. As examples, mannose-class and lactose-class PTS transporters are depicted. Phosphoryl groups are sequentially transferred from PEP to EI, HPr, and subsequently to the transporter subunits IIA. The phosphoryl group is finally transferred from the IIB domain to the incoming sugar. Phosphorylation of HPr by HPrK at Ser(46) is also indicated.

The PTS thus constitutes a phosphoryl-transfer chain that starts at EI (Fig. 1), which can be phosphorylated by PEP at a histidine residue in the presence of Mg^2+^. Phospho-EI transfers the phosphoryl group to HPr, which becomes phosphorylated at a conserved histidine-15 residue [10]. P~His-HPr functions as a phosphoryl donor to the different enzyme II complexes. In *Firmicutes*, HPr can undergo a second ATP-dependent phosphorylation at a serine-46 residue, catalyzed by a metabolically activated HPr kinase (HPrK; see Fig. 1) [11,12]. This ATP-dependent phosphorylation plays a major role in carbon catabolite repression (CCR) in these bacteria [13]. HPrK monomers are constituted by two structural domains: the carboxyl terminal domain displays the kinase and phosphorylase activities and responds to all known effectors similarly as the entire enzyme [14] whereas the function of the N-terminal domain is unknown [15].

The PTS has been thoroughly studied in some *Enterobacteriaceae *and *Firmicutes*. These studies have shown that PTS proteins participate in many other physiological processes such as chemotaxis, regulation of carbon metabolism, coordination of carbon and nitrogen metabolism, and others [3,6,7]. In some bacteria, especially in *Proteobacteria*, a number of paralogs of the general cytoplasmic, energy-coupling EI and HPr proteins are present. Some of these paralogs are apparently specialized in a regulatory role. For example, in *Escherichia coli *paralogs of EI (EI^Ntr^), HPr (NPr) and a fructose-class IIA protein (IIA^Ntr^) constitute a parallel PTS-ptc that apparently functions only in regulation [16]. Furthermore, some PTS proteins interact with other non-PTS proteins modulating their activity [3]. For example, IIA^Glu ^mediates CCR in enterobacteria by interacting with adenylate cyclase together with an additional non-characterized regulatory factor [17]. In *Firmicutes*, P-Ser-HPr acts as a co-regulator of a LacI/GalR type protein named CcpA [18,19], enabling its binding to the *cre *sites preceding catabolite-controlled transcription units [20].

The distribution and evolutionary origin of PTS components have been analyzed in a number of studies [7,21-26] which have suggested that PTS is exclusively found in bacteria. However, a gene cluster which encodes a complete PTS is present in the archaeon *Haloarcula marismortui*, harbored in the plasmid pNG700 [27]. Moreover, the distribution of PTS among different bacteria is very uneven [7] and shows significant differences between the components of the phosphorylation cascade and PTS transporters: some bacteria possess complete PTS-ptc although they lack PTS transporters [7,23]. These data suggest that the genes responsible for the PTS-ptc and the transporters have different evolutionary histories. Furthermore, previous analyses of the constituents of the PTS-ptc indicated that the evolution of these proteins would be best explained by vertical inheritance in some bacterial groups [23] whereas a study of the mannose-class PTS transporters indicated that the evolution of these transporters has been driven primarily by horizontal gene transfer (HGT) [26]. This difference, along with the central role in the regulation of gene expression in some bacterial groups played by PTS-ptc prompted us to study in detail the evolution of PTS elements involved in the phosphorylation cascade (EI, HPr and HPrK).

## Results and discussion

### Genetic organization and distribution of *pts *genes

A total of 222 microbial genomes were screened for genes encoding EI (*ptsI*), HPr (*ptsH*) or HPrK (*ptsK*). This data set included 19 *Archaea *and 153 *Bacteria *species (Table S1, Additional file 1). Eukaryotic genomes were also investigated with negative results, thus confirming the absence of homologs of PTS proteins. The combination of TBLAST and PSI-BLAST searches allowed us to identify all genes and possible pseudogenes encoding the main components of the PTS-ptc. EI and HPr were found either as single polypeptides or as fusion proteins (multiphosphoryl transfer proteins, MTPs), usually together with a IIA domain (Table S2, Additional file 1), whereas HPrK was present as a single polypeptide with the exception of *Fusobacterium nucleatum*, which encodes a fusion protein constituted by two HPrK domains in tandem. In contrast to *ptsK*, which is present in a single copy with the exception of *Oceanobacillus iheyensis*, a varying number of paralogs of *ptsH *and *ptsI *could be found in some species, mostly *Proteobacteria *(Table S2, Additional file 1). For example, *E. coli *K12 harbors five EI-encoding and six HPr-encoding paralogs, either as single polypeptides or as domains of MTPs (Supplementary Table 2).

The distribution of PTS genes has been reviewed recently [7] and we will only consider it briefly here. The components of PTS-ptc (encoded by *ptsH *and *ptsI*) were found in most groups of bacteria included in the data set (Table S1, Additional file 1). Relevant exceptions were early differentiated bacterial groups such as *Aquificales*, *Thermotogales *and the *Thermus/Deinococcus *group. The presence of a PTS cluster in *Deinococcus radiodurans *is not a primitive trait and will be dealt with below. Furthermore, the PTS is absent from *Cyanobacteria *and *Bacteroidetes*. In the remaining groups, the PTS-ptc is incomplete or absent in a few species, mostly obligate intracellular parasites or bacteria with highly specialized lifestyles such as the methanotroph *Methylococcus capsulatus *(γ-*Proteobacteria*), the sulphate-reducing *Desulfotalea psychrophila *(δ-*Proteobacteria*), and the bacterial predator *Bdellovibrio bacteriovorus *(δ-*Proteobacteria*). The absence of these genes in these species from groups where PTS genes are common can be explained by gene losses associated to their specialized lifestyles. This hypothesis agrees with the phylogenetic reconstructions as discussed below.

Our results also showed a strong correlation in the pattern of presence/absence of EI and HPr: only a few species such as *Bartonella *and *Ureaplasma urealyticum *harbored *ptsH *genes while lacking *ptsI *(Table S2, Additional file 1). This correlation was not observed for HPrK, whose distribution is more limited: genes encoding HPrK are absent from *Actinobacteria *and most species of γ-*Proteobacteria*.

The inspection of genome sequences revealed that PTS proteins are absent from *Archaea *with the exception of *Haloarcula marismortui*, which harbors a gene cluster encoding EI, HPr and the IIA, IIB and IIC constituents of a fructose-class PTS transporter in the plasmid pNG700 [27]. To our knowledge, this is the only case of a PTS transporter in *Archaea*.

### Phylogenetic information content of the data sets

EI, HPr and HPrK constituted quite heterogeneous data sets: the EI alignment consisted of 201 sequences and 399 conserved positions after its refinement with Gblocks. Two hundred and four putative homologs of HPr were found in the 222 genomes analyzed. Due to its short length, the initial multiple alignment of HPr was improved only by manual refinement, resulting in a 93-positions alignment. The HPrK alignment had only 77 sequences and 337 positions after manual refinement.

The phylogenetic information content of the sequences was evaluated using two different approaches. Firstly, we carried out a likelihood mapping analysis (Fig. S1, Additional file 2). Briefly, this analysis enables to estimate the suitability for phylogenetic reconstruction of a data set from the proportion of unresolved quartets in a maximum likelihood analysis. As expected from the low number of positions in its alignment, HPr was the protein with the lowest phylogenetic content (only 59.4% fully resolved quartets). In contrast, both EI and HPrK had higher phylogenetic information contents, with 88.4% and 86.8% fully resolved quartets, respectively. Secondly, split networks were obtained using the Neighbor-net algorithm implemented in Splitstree. The resulting network contained not only the maximum-likelihood topology but also additional splits not reflected in the phylogenetic tree.

The networks derived from the three proteins (data not shown; available upon request) showed considerable conflicting signal, particularly in the HPr network. In contrast, the HPrK network showed a clearer tree-like structure with better defined deep relationships, which will be discussed later along with the corresponding phylogenetic tree. Finally, the EI network presented a mixed picture. On the one hand, several proteins presented no clear relationships to any of the main groups. On the other hand, network analyses limited to specific groups revealed that conflicting signals concentrated in the most internal nodes. As a whole, the three genes presented unresolved deep phylogenetic relationships along with a number of well defined groupings and lastly, a few taxa with no clear phylogenetic relationships.

### Phylogenetic reconstruction

**Figure 2:**
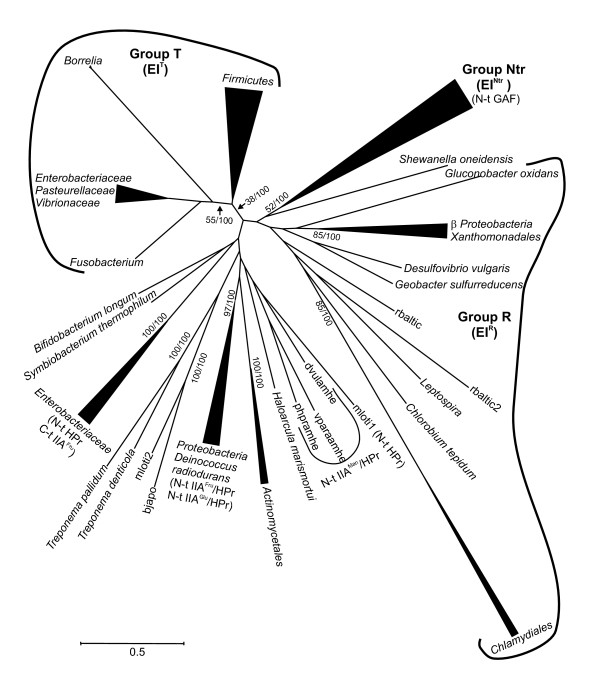
**Summarized maximum likelihood topology of the EI sequences used in this study**. The complete tree is shown in Fig. S2, Additional file 2. Support values for the bootstrap analysis by maximum likelihood (BS) and for Bayesian *posterior *probabilities (BPP) are given for those nodes with BS > 50% or BPP = 1.00. The main groups derived from the analysis are indicated as well as the domain composition of MTPs. Sequences rbaltic and rbaltic2 correspond to *Rhodopirellula baltica*; mloti1 and mloti2, *Mesorhizobium loti*; dvulamhe, *Desulfovibrio vulgaris *[GenBank:YP_010202]; vparaamhe, *Vibrio parahaemolyticus*; phpramhe, *Photobacterium profundum*; bjapo, *Bradyrhizobium japonicum*. Additional details are provided in Table S2, Additional file 1.

For the three alignments, the best evolutionary model under the AIC criterion was rtREV [28], a model originally developed from the analysis of retroviral sequences but which has been shown to be one of the most commonly retrieved models for bacterial sequence alignments [29]. The large phylogenetic distances encompassed in the three alignments resulted in the absence of a significant fraction of invariant sites, and in consequence only the gamma distribution was used to take heterogeneities in evolutionary rates among sites into account. Although maximum likelihood and Bayesian topologies were generally coincident there were also some remarkable differences in support values for nodes. For instance, some groups were clearly better resolved in the Bayesian than in the maximum likelihood topology. This is exemplified in the EI topology (Fig. 2 and Fig. S2, Additional file 2) where larger groups like EI^Ntr ^(bootstrap support, BS = 52%; Bayesian posterior probability, BPP = 1.00), T (BS = 38%, BPP = 1.00) and R (BS = 25%, BPP = 0.79) were statistically supported only by the Bayesian analyses.

The EI topology was characterized by the low support of its most internal nodes. Nevertheless, three large groups could be delineated. Although none of these groups had a bootstrap support value larger than 50% (Fig. 2 and Fig. S2, Additional file 2), the bayesian analysis, genomic context analyses and the function of some of the corresponding products justify their use as units for analysis in this work (see below). The first group (group Ntr; gene *ptsP*, EI^Ntr^) is monophyletic and it consisted of genes from α- and γ-*Proteobacteria *and *Geobacter sulfurreducens *(δ-*Proteobacteria*) encoding EI proteins with an N-terminal GAF domain. The second group (group R) encompassed *ptsI *genes (denoted EI^R ^to distinguish them from other EI) present in organisms that lack PTS transporters or harbor additional EI-encoding paralogs (mostly MTPs) clustered with genes encoding PTS transporters. The third group (T) consisted mainly of *ptsI *genes from *Firmicutes *and γ-*Proteobacteria *(EI^T^). Finally, a number of smaller groups which encompassed mostly *ptsI *genes of *Actinomycetes *and genes encoding MTPs could be delineated (Fig. 2).

**Figure 3:**
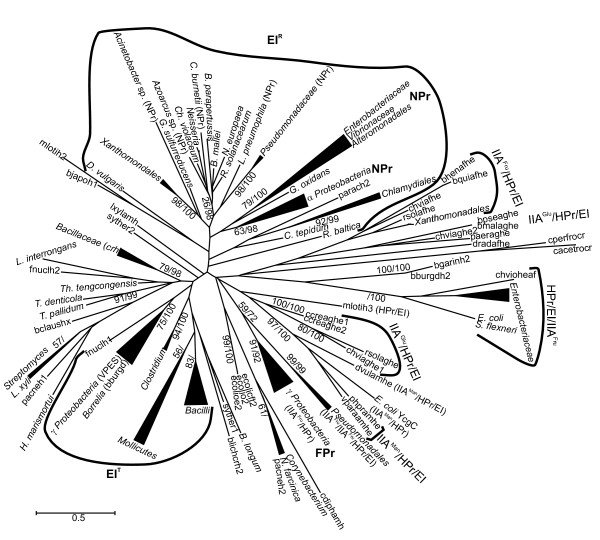
**Summarized maximum likelihood topology of the HPr sequences used in this study**. The complete tree is shown in Fig. S3, Additional file 2. Support values for the bootstrap analysis by maximum likelihood and for Bayesian posterior probabilities are given as in Figure 2. Clusters of orthologous sequences to *E. coli *FPr and NPr are indicated. Groups of MTPs and clusters of sequences functionally related to EI groups R, Ntr and T are indicated by surrounding lines. The domain composition of MTPs is also indicated. Sequence parach2 corresponds to Candidatus *Protochlamydia amoebophila*; bhenafhe, *Bartonella henselae*; bquiafhe, *Bartonella quintana*; chviafhe, chviaghe1, chviaghe2 and chvioheaf, *Chromobacterium violaceum*; rsolafhe and rsolaghe, *Ralstonia solanacearum*; bpseaghe, *Burkholderia pseudomallei*; bmalaghe, *Burkholderia mallei*; paeraghe, *Pseudomonas aeruginosa*; dradafhe, *Deinococcus radiodurans*; cperfrocr, *Clostridium perfringens*; cacetrocr, *Clostridium acetobutylicum*; bgarinh2, *Borrelia garinii*; bburgdh2, *Borrelia burgdorferi*; mlotih2 and mlotih3, *Mesorhizobium loti*; ccreaghe1 and ccreaghe2, *Caulobacter crescentus*; pacneh1 and pacneh2, *Propionibacterium acnes*; ecolicft2, ecolio2 and ecolioe2, *Escherichia coli*; syther1 and syther2, *Symbiobacterium thermophilum*; blichcrh2, *Bacillus licheniformis*; fnuclh1 and fnuclh2, *Fusobacterium nucleatum*; bclaushx, *Bacillus clausii*; lxylamh, *Leifsonia xyli*; bjapoh1, *Bradyrhizobium japonicum*. Additional details are provided in Table S2, Additional file 1.

In agreement with its low phylogenetic information content, the HPr tree (Fig. 3 and Fig. S3, Additional file 2) had high support values only in some external nodes. For example, the HPr paralogs associated to fructose transport (FPr) form a well-supported monophyletic group (BS = 91%; BPP = 0.92) of sequences from *Enterobacteriaceae*, *Pasteurellaceae *and *Vibrionaceae*. Therefore, on its own, this phylogenetic reconstruction cannot be taken as a reliable reflection of the evolutionary history of these genes and it was not possible to analyze the concordance/congruence between the larger groups described above for the EI topology and their HPr counterparts. However, it is worth mentioning that there was a good agreement between the small, well-supported groups at the tips of the HPr tree and their corresponding EI counterparts. The analysis of their genomic context also supported the evolution of EI and HPr as a single unit, as we will detail below.

**Figure 4:**
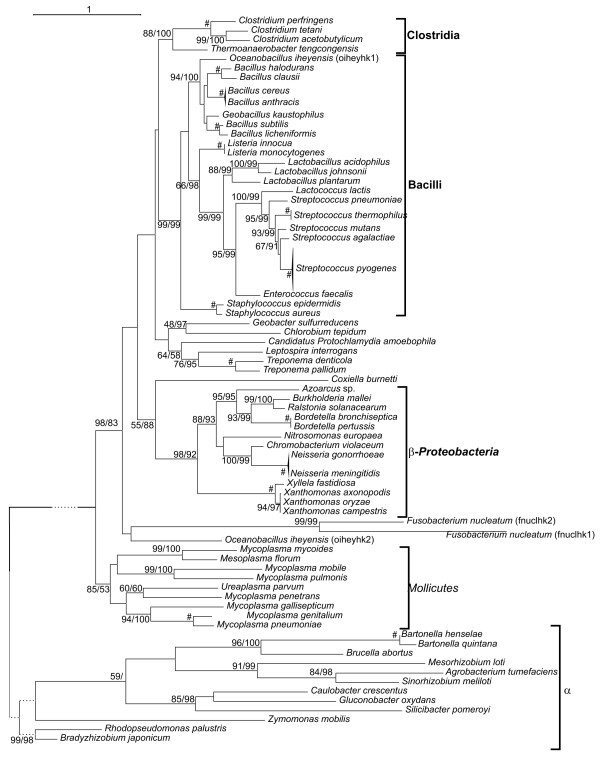
**Maximum likelihood topology of the HprK sequences used in this study**. The tree is arbitrarily rooted with α-*Proteobacteria*. Support of nodes is indicated as in Figure 2. # indicates nodes with BS = 100% bootstrap and BPP = 1.00. The length of the α-*Proteobacteria *branch has been shortened (indicated by dotted lines).

The phylogenetic analysis of HPrK proteins was complicated by the presence of truncated proteins in α-*Proteobacteria *and *Coxiella burnetti*. These proteins have preserved only the C-terminal domain of HPrK and introduced numerous gaps in the alignment. α-*Proteobacteria *sequences appeared as a long branch stemming from the base of the *Mollicutes *cluster (Fig. 4). β-*Proteobacteria *(including the HPrK sequence of *C. burnetti*, a γ-*Proteobacteria*) also constituted a distinct group, although their position did not reveal a clear relationship with α-*Proteobacteria *(Fig. 4). On the other hand, sequences from *Firmicutes *were arranged in three distinct monophyletic clusters corresponding to *Bacilli*, *Clostridia *and *Mollicutes *(Fig. 4), with the exception of a second *ptsK *homolog encoded by *Oceanobacillus iheyensis *(oiehyk2, Fig. 4). The last group was constituted by a paraphyletic cluster which included Spirochetes, Candidatus *Protochlamydia amoebophila*, *Chlorobium tepidum *and *G. sulfurreducens*, a δ-*Proteobacteria*.

### Genomic context and functional information

The phylogenetic analyses of the different constituents of the PTS-ptc were not sufficient, on their own, to explain the evolutionary history of this system. Therefore, we considered the genomic context and the functional information available in order to better interpret the previous phylogenetic results. The functional information available, the distribution of PTS transporters in different species and the genome context showed a noteworthy correspondence with the groups observed in the phylogenetic reconstruction of EI. Therefore, we will start by discussing the phylogenetic relationships derived from EI proteins in relation to the information available on their functional role and the genomic context of the corresponding genes while HPr and HPrK will be discussed subsequently in relation to these results.

### The evolutionary history of the regulatory PTS-ptc: EI^Ntr ^and EI^R ^are functional analogs

**Figure 5:**
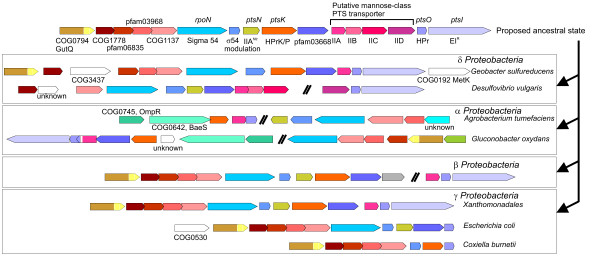
**Schematic representation of the proposed ancestral *rpoN *gene cluster of *Proteobacteria***. Representative clusters of the major divisions of *Proteobacteria *are depicted (additional details are shown in Fig. S4, Additional file 2).

Group R genes were found in *Proteobacteria *and the related taxa *Chlamydiales*, *Chlorobia*, *Planctomycetes *and the spirochaete *Leptospira interrogans *(Fig. 2). With the exception of some *Proteobacteria*, none of these organisms harbors PTS transporters [7]. Although group R was not well supported in the phylogenetic analysis (EI^R^; Fig. 2, BS = 25%, BPP = 0.79), the similarity in their corresponding gene clusters (Fig. 5 and Fig. S4, Additional file 2) and the topological congruence between the EI phylogeny for group R and the 16S rRNA tree (p = 0.522 in the Shimodaira-Hasegawa test; Fig. S5A and S6, Additional file 2) suggest that the gene encoding EI^R ^was present in the last common ancestor of *Proteobacteria *and, possibly, in that of *Chlamydiales*, *Chlorobia*, *Planctomycetes *and *Proteobacteria*.

Genes belonging to the Ntr group (*ptsP *genes) are found exclusively in *Proteobacteria *and they encode EI^Ntr ^[30]. The phylogenetic analysis showed good support (BS = 52%, BPP = 1.00) and congruence between the branching order of EI^Ntr ^encoding genes and the expected organismal descent according to the 16S rRNA phylogenetic tree (p = 0.363 in the Shimodaira-Hasegawa test; Fig. S5B and S6, Additional file 2). These results suggest that this gene evolved at an early stage in the evolutionary history of *Proteobacteria*, possibly predating the differentiation of δ-*Proteobacteria *(the most basal group in this clade).

In *Enterobacteriaceae*, and possibly in other *Proteobacteria *as well, EI^Ntr ^constitutes a phosphorylation chain together with the product of *ptsO *(encoding an HPr paralog referred to as NPr) and the product of *ptsN *(IIA^Ntr^). Interestingly, the phylogenetic analysis of HPr-encoding genes showed that NPr sequences of α- and γ-*Proteobacteria *cluster together with HPr sequences of β-*Proteobacteria*, δ-*Proteobacteria *and *Xanthomonadales *(Fig. 3 and Fig. S3, Additional file 2).

The inspection of the corresponding gene clusters also points to a close relationship between NPr- and HPr-encoding genes associated to EI^R^. In *Xanthomonadales*, *ptsH *and *ptsI *(encoding EI^R^) constitute an operon together with a gene encoding a IIA^Man ^protein and next to the cluster of *rpoN *genes, which includes a *ptsN *homolog and *ptsK*. Similar gene clusters encompassing *ptsN*, *ptsO *and *rpoN *are also present in the other proteobacterial groups (Fig. 5 and Fig. S4, Additional file 2).

From the results of phylogenetic reconstruction and cluster composition analyses we hypothesize that a cluster encompassing *rpoN*, *ptsK*, *ptsI*, *ptsO *and *ptsN *genes, among others, was assembled at an early stage in the evolution of *Proteobacteria *(Fig. 5 and Fig. S4, Additional file 2). Subsequent gene rearrangements led to the clusters observed in extant species (Fig. 5 and Fig. S4, Additional file 2). These rearrangements included the loss of *ptsI *(EI^R^) in most α- and γ-*Proteobacteria *and the loss of *ptsK *and the gene encoding IIA^Man ^in γ-*Proteobacteria*, with the exception of *Xanthomonadales *which conserved both genes and *C. burnetti *which only conserved a *ptsK *gene lacking the N-terminal domain.

Considering the observed current distribution of *ptsI *(EI^R^) and *ptsP *(EI^Ntr^) genes and the congruence of the topologies of both groups with the accepted branching order observed in the 16S rRNA tree (Fig. S6, Additional file 2) we hypothesize that *ptsI *(EI^R^) and *ptsP *were present in the last common ancestor of α-, β-, δ- and γ-*Proteobacteria*, a composition still found in *G. sulfurreducens*, and their current distribution is explained by vertical inheritance and lineage-specific gene losses. Along the evolution of the different proteobacterial lineages only one of these genes was preserved: *ptsI *(EI^R^) was lost in α-*Proteobacteria*, except in *Gluconobacter oxidans *(the *ptsI *genes present in *Mesorhizobium loti *and *Bradyrhizobium japonicum *are not orthologous and they will be discussed separately), and the same occurred in γ-*Proteobacteria*, with the exception of *Xanthomonadales*. However, in *G. oxidans*, β-*Proteobacteria *and *Xanthomonadales*, the lost gene was *ptsP*.

There is no functional information on the role of EI^R ^although it has been proposed to participate, along with HPr and HPrK, in a phosphoryl transfer chain involved in the σ^54 ^regulon [15]. Our analysis agrees with this proposal and provides an explanation for the displacement of EI^R ^by EI^Ntr ^in some proteobacterial lineages. Indeed, there are evidences indicating that EI^Ntr ^is involved in regulating the expression of genes belonging to the σ^54 ^regulon both in α-*Proteobacteria *[31] and γ-*Proteobacteria *[16,32].

In consequence, we hypothesize that EI^R ^and EI^Ntr ^are functional analogs that play a regulatory role. However, the presence of genes encoding a putative mannose-class PTS transporter in *D. vulgaris *(see Fig. 5 and Fig. S4, Additional file 2) hints at the possibility that EI^R ^also plays a role in sugar transport [33]. Furthermore, a phylogenetic reconstruction of IIA^Man ^encoding genes shows that the sequence of *D. vulgaris*, which is part of the putative PTS transporter, clusters together with other IIA^Man ^encoding genes from α- and β-*Proteobacteria *located in the *rpoN *gene cluster (data not shown). Therefore, these genes may have been present in the ancestral proteobacterial cluster and the genes encoding subunits IIB, IIC and IID were subsequently lost in most proteobacterial lineages.

### The PTS-ptc of *Actinobacteria*

The phylogenetic analysis of *ptsI *genes from *Actinobacteria *shows that those genes from species belonging to the order *Actinomycetales *constitute a monophyletic group with strong support (Fig. 2). This clustering is not supported in the *ptsH *tree possibly due to its poor resolution (Fig. 3). As a difference, *ptsI *and *ptsH *of *Bifidobacterium longum *and *Symbiobacterium thermophilum *cluster separately from those of other *Actinobacteria *(Fig. 2 and 3). Since the observed topology of the *ptsI *tree is congruent with the expected order of organismal descent (Fig. S6, Additional file 2) and both genes are present in most actinobacterial species included in this study, it is likely that both genes were present in the last common ancestor of *Actinomycetales *and at least *ptsI *was inherited vertically.

However, the analysis of the genomic context shows different gene organizations and points towards possible duplications or HGT events among actinobacterial species which would have involved HPr (Fig. S7, Additional file 2). For example, *Propionibacterium acnes *harbors a *ptsH *gene (pacneh2) which clusters with homologs from *Nocardia *and Corynebacteria and it is located in a *deoR-fruK-fruA-ptsH *operon identical to those found in these species (Fig. 3 and Fig. S7, Additional file 2), whereas its paralog (pacneh1) clusters with its counterparts of *Streptomyces *and *Leifsonia xyli*. Another example is given by the fusion proteins encoded by *L. xyli *and *Corynebacterium diphtheriae *which consist of a IIA^Man ^domain and a C-terminal HPr domain (sequences cdiphtamh and lxylamh) located in identical operons together with genes encoding dihydroxyacetone kinase (Fig. S7, Additional file 2). Nevertheless, these sequences appear very distantly related to each other and to similar proteins of *Proteobacteria *in the HPr tree (Fig. 3). In summary, these results suggest that HGT or lineage-specific duplications affecting *ptsH *have occurred in *Actinobacteria *but currently available data are insufficient to ascertain this point.

### A PTS system in *Archaea*

**Figure 6:**
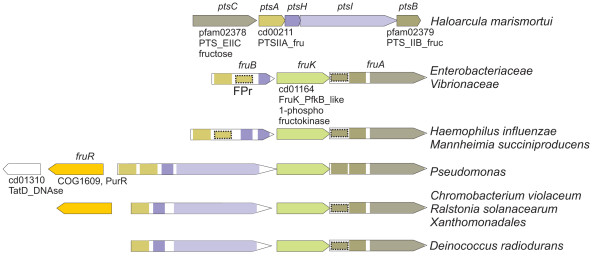
**Schematic representation of selected clusters containing genes encoding FPr and related fructose class PTS proteins**. Colors indicate homologous genes or domains. Dashed rectangles indicate possibly non-functional homologous domains (additional details are shown in Fig. S8, Additional file 2).

*H. marismortui *harbors in plasmid pNG700 a gene cluster encoding a fructose-class PTS transporter, EI and HPr (Fig. 6). Nevertheless, neither the phylogenetic analyses of EI and HPr nor the structure of the cluster provided evidence for a clear relationship to any of their bacterial counterparts. In contrast to other fructose PTS gene clusters discussed below, EI and HPr do not constitute a MTP; likewise, the constituents of the transporter are not encoded by fusion genes, in contrast to that found in the other fructose clusters studied here (see Fig. 6 and Fig. S8, Additional file 2). The phylogenetic analysis placed *H. marismortui *EI as a basal branch of MTPs containing IIA^Man ^domains (Fig. 2) whereas HPr clustered with actinobacterial sequences. Furthermore, the lengths of the branches of these genes in their corresponding trees are comparable to those of their bacterial counterparts and far shorter than it would be expected for archaeal genes (see for example, Fig. S6, Additional file 2). Therefore, although the phylogenetic analyses and the uniqueness of this operon in *Archaea *indicate that this operon is of bacterial origin, the currently available data do not allow a more precise identification of its source.

#### MTPs containing IIA^Fru ^or IIA^Glu ^domains

MTPs can be divided into three main groups: proteins containing N-terminal IIA^Fru ^or IIA^Glu ^domains, proteins containing IIA^Man ^N-terminal domains and proteins containing C-terminal IIA^Fru ^domains. MTPs containing one or two N-terminal IIA^Fru ^domains (encoded by *fruB*) are typically associated to fructose-class PTS transporters (encoded by *fruA*) and 1-phospho fructokinases (*fruK*; see Fig. 6 and Fig. S8, Additional file 2). The functional information available from *E. coli *[34], *Rhodobacter capsulatus *[35], *Salmonella typhimurium *[36] and *Xanthomonas campestris *[37,38] indicates that all these transporters internalize fructose.

The prototypic FruB proteins (usually termed FPr) of *Enterobacteriaceae *and *Vibrionaceae *are composed of a IIA^Fru ^domain separated by an intervening domain from a C-terminal HPr domain (Fig. 6 and Fig. S8, Additional file 2). In *Pasteurellaceae*, these proteins have undergone some modifications: FPr proteins from *Haemophilus influenzae *and *Mannheimia succiniproducens *carry duplicated HPr domains which possibly resulted from a relatively recent duplication [39]. In *Pseudomonas*, FruB proteins consist of two IIA^Fru ^domains arranged in tandem and fused to a central HPr domain and a C-terminal EI domain (Fig. 6 and Fig. S8, Additional file 2). This second IIA^Fru ^domain (IIA^Fru'^) is apparently absent in the closely related *Acinetobacter *sp. However, the alignment of enterobacterial FPr proteins together with those of *Pasteurellaceae*, *Pseudomonas *and *Acinetobacter *reveals that the intervening domain is in fact a heavily altered, remnant of the IIA^Fru' ^domain (Fig. S9, Additional file 2). Interestingly, the cognate FruA transporters also possess duplicated IIB domains even in those organisms whose phosphoryl transfer proteins carry only one functional IIA^Fru ^domain [37,40]. At least in *E. coli *both IIB domains are required for full activity [34].

The phylogenetic reconstructions of the EI domains of these proteins along with the structure of the *fru *gene clusters strongly suggest that all FruB evolved from a common ancestor consisting of duplicated IIA domains fused to HPr and EI domains. This ancestral form is conserved in *Pseudomonas*. FruB genes harboured by *Chromobacterium violaceum*, *D. radiodurans*, *Ralstonia solanacearum *and *Xanthomonas *probably evolved from a common ancestor where the IIA^Fru' ^domain was removed. Finally, the EI domain was lost in *Enterobacteriaceae*, *Pasteurellaceae *and *Vibrionaceae *giving rise to FPr. Reasons for this proposal will be discussed in the section dedicated to group T. The phylogenetic relationships inferred from the sequences of FPr proteins are in agreement with the most accepted order of organismal descent represented by the 16S rRNA phylogeny (Fig. S6, Additional file 2). Therefore it is reasonable to assume that the *fru *operon was present in the last common ancestor of these families and FruB was modified to its current structure before the differentiation of these lineages.

Similarly, the phylogenetic position of proteins containing IIA^Glu ^N-terminal domains suggests that they evolved from a FruB ancestor by substitution of the IIA^Fru ^domain by a IIA^Glu ^domain. This hypothesis is based on their branching within the FruB group (Fig. 2 and Fig. S2, Additional file 2).

In summary, the phylogenetic analysis does not allow establishing a clear origin for MTPs containing N-terminal IIA^Fru ^or IIA^Glu ^domains. The strong support for this cluster (BS = 97%; BPP = 1.00; see Fig. 2) indicates that they originated from a common ancestor and, since most MTPs are harbored by *Proteobacteria*, it seems reasonable to hypothesize that these genes evolved from a proteobacterial ancestor. However, the phylogenetic analyses do not provide evidence for a close relationship of either the EI or the HPr domains of MTPs containing N-terminal IIA^Fru ^or IIA^Glu ^domains to any other EI or HPr paralogs present in *Proteobacteria*.

MTPs with a IIA^Fru ^C-terminal domain constitute a monophyletic group with strong support in both EI and HPr trees and with no clear phylogenetic relationship to other MTPs. Hence, they were possibly assembled independently of MTPs containing N-terminal IIA domains.

### MTPs containing IIA^Man ^domains

**Figure 7:**
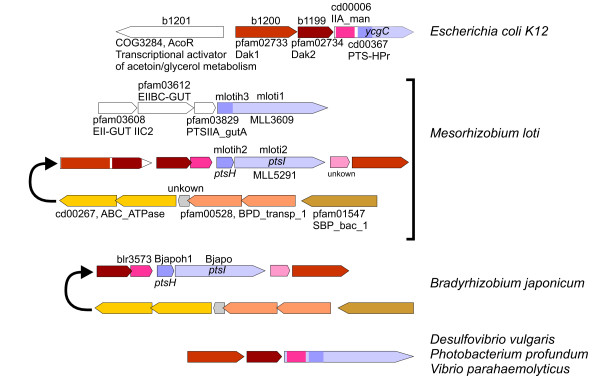
**Gene clusters containing *ptsH *or *ptsI *associated to genes encoding dihydroxyacetone kinases and phylogenetically related clusters**. Colors indicate homologous genes or domains.

MTPs containing IIA^Man ^domains are invariably associated to genes encoding dihydroxyacetone kinases (Fig. 7). This group also includes the YcgC proteins present in *E. coli *strains which lack the EI domain [41]. The phylogenetic analysis of HPr domains showed strong support for this group (BS = 97%; BPP = 1.00; Fig. 3) and indicated that these proteins evolved from a common ancestor.

The phylogenetic reconstruction of *ptsI *sequences shows that a gene encoding a fusion protein consisting of an HPr N-terminal domain and an EI C-terminal domain from *Mesorhizobium loti *(sequences mlotih3 and mloti1, HPr and EI domains, respectively) clusters with this group. This gene is located in an operon together with a cluster of genes encoding a glucitol-class PTS transporter (Fig. 7). It is feasible that the MTP present in the glucitol-class PTS evolved from an ancestor containing a IIA^Man ^domain which was subsequently lost. Interestingly, *M. loti *encodes a second EI (mloti2) which constitutes an operon with an HPr encoding gene (mlotih2) and genes encoding dihydroxyacetone kinase. An identical cluster is present in *Bradyrhizobium japonicum *(bjapo and bjapoh1; Fig. 7). None of the EI and HPr present in these clusters grouped with IIA^Man ^MTPs. In order to determine whether these sequences had a significantly accelerated evolutionary rate in comparison to MTPs containing IIA^Man ^domains, a Tajima's relative rate test was carried out by comparing sequences mloti1, mloti2 and bjapo with that of *Vibrio parahaemolyticus *(vparaamhe) using an *E. coli *MTP with a C-terminal IIA^Fru ^domain (ecolcheag) as an outgroup. The test revealed that none of these sequences were significantly accelerated (p = 0. 346, p = 0.448 and p = 0.206, for mloti1, mloti2 and bjapo, respectively). Therefore, the origin of the *ptsH *and *ptsI *genes present in the dihydroxyacetone kinase-encoding clusters of *M. loti *and *B. japonicum *cannot be further elucidated from the available data.

### The evolution of group T: vertical inheritance in *Firmicutes *and transference to γ-*Proteobacteria*

The T group (EI^T^) includes all *Firmicutes *sequences, *F. nucleatum*, *Borrelia *and EI encoding genes from *Enterobacteriaceae*, *Pasteurellaceae *and *Vibrionaceae*. This group had low bootstrap support although it was strongly supported in the Bayesian analysis (BS = 38%, BPP = 1.00). The phylogenetic analysis of *ptsI *indicates that *Firmicutes *constitute a monophyletic group although with low support in some branches (Fig. S2, Additional file 2). The splits network (not shown, available upon request) also indicated conflicting signals in this group deriving from the difficulties in determining the phylogenetic relationships among *Bacilli*, *Clostridia *and *Mollicutes*. Despite the presence of incongruence, the monophyly of *Firmicutes *is also observed in the *ptsH *topology. Furthermore, the phylogenetic reconstruction of EI^T ^of *Firmicutes *agrees with the expected order of organismal descent for this group (Fig. S6, Additional file 2). Taken together, these data suggest that *ptsH *and *ptsI *evolved as a single unit in *Firmicutes*, as reflected by the agreement of both topologies, and that vertical inheritance has been their primary mode of transmission.

**Figure 8:**
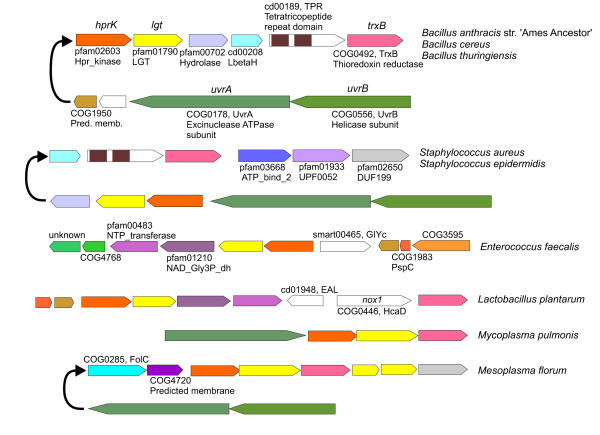
**Selected gene clusters containing *ptsK *present in *Firmicutes***. Colors indicate homologous genes or domains (additional details are shown in Supplementary Figs. S10 and S11, Additional file 2).

In contrast, the *ptsK *sequences of *Clostridiales *and *Bacillales *cluster together in the phylogenetic whereas *Mollicutes *form a distinct cluster together with *ptsK *sequences of α-*Proteobacteria *(Fig. 4). Nevertheless, the inspection of *ptsK *gene clusters suggests a closer relationship between *Mollicutes *and other *Firmicutes *(Fig. 8 and Figs. S10 and S11, Additional file 2). In *Bacillales *and *Lactobacillales*, *ptsK *is always found with *lgt*, an arrangement also found in *Mollicutes*. Moreover, the common occurrence of *ptsK *together with *uvrA *and *uvrB *on one hand and *trxB *on the other observed in *Bacillales *is also found in many *Mollicutes*.

**Figure 9:**
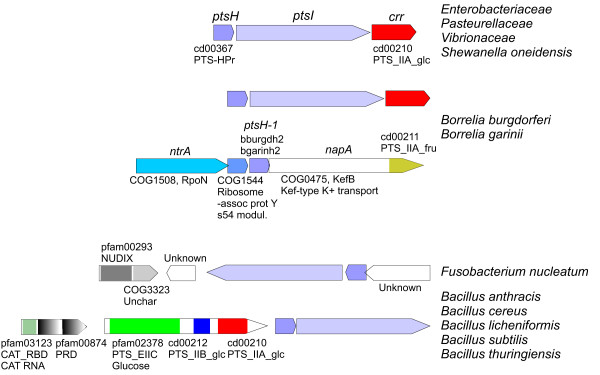
**Selected clusters containing genes encoding EI^T^**. Colors indicate homologous genes or domains (additional details are shown in Fig. S12, Additional file 2).

Group T genes are also present in a limited number of γ-*Proteobacteria *families. In addition, the analysis of HPr sequences provides evidence for a close relationship of *Shewanella oneidensis ptsH *to this group (Fig. S3, Additional file 2) although the corresponding EI sequence did not cluster within the T group (Fig. 2). On the other hand, *S. oneidensis *harbors a *ptsHIcrr *operon identical to those present in group T γ-*Proteobacteria *(Fig. 9 and Fig. S12, Additional file 2). In order to better understand the basis of this discrepancy a Tajima's relative rate test was carried out by comparing the sequence of *S. oneidensis *(soneid) with that of *Vibrio cholerae *(vcholer) using *F. nucleatum *(fnucleat) as an outgroup. The test revealed that this sequence (soneid) is significantly accelerated (p < 0.001) with respect to their enterobacterial counterparts, a phenomenon that usually results in unexpected, and incorrect, positions in phylogenetic reconstructions. Therefore, the *ptsI *gene of *S. oneidensis *possibly shares the same origin than other proteobacterial EI^T ^encoding genes although it has diverged extremely. In summary, phylogenetic and gene content analyses suggest that HPr and EI^T ^from *Vibrionaceae*, *Pasteurellaceae*, *Enterobacteriaceae *and *Shewanella *(hereafter called VPES group) evolved from a common ancestor.

Phylogenetic trees show that VPES sequences cluster together with *Fusobacterium nucleatum *and *Borrelia *sequences as their closest and basal relatives. This group forms a sister clade with *Firmicutes *in both EI and HPr trees (Figs. 2 and 3 and Figs. S2 and S3, Additional file 2) and the network analyses also provided evidence for a close relationship of these genes. The topology of the tree of EI^T ^sequences of VPES as an ingroup was compatible with the order of organismal descent inferred from the 16S rRNA (p = 0.437 in the Shimodaira-Hasegawa test; Fig. S5C, Additional file 2). Discrepancies between the two trees were limited to the positions of some species within the same family.

The observed clustering of genes from such distant lineages can be explained either by an ancestral duplication of EI and HPr in their last common ancestor and subsequent loss in all intervening lineages or by a transfer from *Firmicutes *or *Fusobacteria *to the last common ancestor of VPES. We did not observe significant differences in nucleotide composition or codon usage in EI^T ^sequences from VPES (results not shown) and, as far as we know, these genes have not been detected as horizontally transferred by other researchers [42,43], thus favoring the hypothesis of an ancestral duplication. However, a HGT event is the most parsimonious explanation since it requires a single gain in the last common ancestor of the VPES group whereas the alternative scenario requires multiple, independent losses in other lineages. In this sense, it is worth noting that the group VPES coincides with the superorder lower γ-*Proteobacteria *proposed by Jensen and collaborators [44] further substantiating the idea that VPES constitute a monophyletic group within γ-*Proteobacteria*. The failure to detect *ptsH *or *ptsI *as horizontally transferred to VPES by parametric methods can be explained by amelioration [45,46] since the putative HGT event would have occurred before the differentiation of the VPES lineages. In summary, the phylogenetic analyses and the observed distribution of EI^T ^and HPr-encoding genes suggest that these genes were transferred to the last common ancestor of VPES from *Fusobacteria *or *Firmicutes*.

On the other hand, *Borrelia *species harbor identical *ptsH-ptsI-ccr *operons to the ones found in VPES (Fig. 9 and Fig. S12, Additional file 2). Furthermore, *Borrelia *possesses additional *ptsH *genes (sequences bburgdh2 and bgarinh2) which are very distantly related to their counterparts and are located in gene clusters together with *rpoN *(Fig. 9). The presence of this second *ptsH *gene in a gene cluster similar to that found in other *Spirochaetales*, the absence of the *ptsH-ptsI-ccr *operon in other spirochaetes and the phylogenetic positions of both *ptsH *and *ptsI *close to their VPES counterparts suggest that *Borrelia *acquired this operon via HGT from VPES.

The acquisition of the *ptsH-ptsI *operon by VPES possibly propelled a number of evolutionary novelties in this group. Members of VPES harbor the largest number of PTS transporters in their genomes among *Proteobacteria *[7]. Phylogenetic analyses of some of these transporters have revealed extensive gene exchanges between VPES and *Firmicutes *[26]. Furthermore, some MTPs are in a process of reduction in VPES. As indicated above, FPr and YcgC evolved from proteins which contained EI domains. We hypothesize that the acquisition of the *ptsH *and *ptsI *genes enabled the expansion of PTS transporters by HGT and/or duplication and differentiation, and it also made the EI domains of some MTPs dispensable as EI^T ^took over their function. Most relevantly, EI^T ^and HPr also participate in CCR in *Firmicutes *and VPES. Interestingly, CCR in VPES differs markedly from other closely related proteobacterial groups such as *Pseudomonadales *[47,48]. Moreover, VPES encode a particular type of adenylate cyclase not related to other adenylate cyclases [49] and which is only found in a few species outside this group. Hence, we hypothesize that the transfer of EI^T ^and its cognate HPr-encoding genes was a key event in the appearance of a novel CCR pathway, an evolutionary novelty only shared by the VPES group.

Our hypothesis does not contradict the complexity hypothesis which proposes that genes have different probabilities of being transferred depending on how many interacting partners their products have in the cell [50]. The analysis of the metabolic network of *E. coli *has shown that the success of a HGT event depends on the pathway affected, so that laterally transferred genes that intervene in a peripheral pathway [51] or having physiologically interacting partners already present in the receptor genome [52] are more likely to be fixed. However, the complexity hypothesis does not preclude that a HGT-acquired peripheral pathway might evolve into a complex regulatory network. In our view, the EI^T ^phosphoryl transfer chain would originally have a limited role in glucose transport in VPES. However, the great plasticity of the EI^T^-HPr phosphoryl transfer chain would facilitate the expansion of a regulatory network in an environment where PTS-ptc networks already existed. In this sense it is worth noting that EI^T ^and HPr can phosphorylate NPr and IIA^Ntr ^whereas EI^Ntr ^and NPr cannot replace EI^T ^[6].

## Conclusion

Phylogenetic analyses were not sufficient to reliably disentangle the deepest relationships among the PTS-ptc genes present in extant organisms. Nevertheless, the complementation of phylogenetic analyses with functional information and analyses of the genomic context has allowed us to derive several conclusions.

Our results have provided evidence for an early origin of the PTS-ptc in *Bacteria *and have shown that, while in some lineages such as *Firmicutes *this system has undergone very few modifications, in other lineages, such as *Proteobacteria*, major changes have occurred including the displacement of EI^R ^by EI^Ntr ^in some lineages and the acquisition of a PTS-ptc from *Fusobacteria *or *Firmicutes *by an ancestor of VPES which drove a number of evolutionary novelties in these bacteria. We consider that this is an excellent example illustrating that horizontally transferred components of peripheral metabolic pathways can evolve into complex regulatory networks in the physiological core of bacteria.

## Methods

### Sequences

We have analyzed 222 complete genomes from 172 different prokaryotic species (see Table S1, Additional file 1) retrieved from the NCBI repository (on October 2004). EI, HPr, and HprK encoding genes were retrieved from whole genomes by using PSI-BLAST and TBLASTN [53,54]. The PSI-BLAST searches were performed against the GenBank database using the *E. coli *EI and HPr sequences (Acc. N° NP_416911 and NP_416910, respectively) and the *Bacillus subtilis *HPrK sequence (Acc. N° NP_391380) as query sequences with default settings. The searches were iterated until convergence was attained. From all the sequences retrieved, only those from whole genomes were selected, and these sequences were subsequently filtered attending to the presence of their characteristic domains (pfam02896, pfam05524, and pfam00391 for EI; pfam00381 for HPr and pfam07475 for HPrK) or a coverage of at least 75% of the query sequence. Subsequently, additional PSI-BLAST searches using the most distant selected sequences were performed in order to retrieve possible homologs not identified in the first search. Finally, TBLASTN searches were performed against the available complete genome sequences using the same query sequences as for the PSI-BLAST searches. The corresponding 16S rRNA sequences from each genome were also downloaded. Detailed information on the sequences used in these analyses, accession numbers, and domain structure is provided in Table S2, Additional file 1. In a few cases, possible frame-shifts in putative coding sequences were corrected (see Table S2, Additional file 1), and the translated products were used in this study. The data sets were refined by excluding redundant sequences.

### Alignment and phylogenetic information analysis

Multiple alignments were obtained with ClustalW [55] and manually corrected where necessary. Positions of doubtful homology or introducing phylogenetic noise due to an excessive number of gaps were removed using Gblocks [56] with default settings for the EI alignment. However, due to the short length of HPr and HPrK sequences we decided to apply only a manual refinement to the corresponding alignments in order to keep a maximum number of positions for analysis. The final multiple alignments used for the analyses are available from the authors upon request.

The phylogenetic signal contained in the different data sets was assessed by likelihood mapping [57] using Tree-Puzzle 5.2 [58] with the JTT model [59] of amino acid evolution and a discrete gamma distribution to account for heterogeneity in evolutionary rates among positions in the multiple alignments. In addition, a phylogenetic network of each protein was reconstructed using the JTT model to compute pairwise distances and the neighbor-net algorithm [60]. Splitstree4 [61] was used to represent the networks. This network analysis allows screening for possible non tree-like evolutionary events and the influence of phylogenetic noise [62].

Functional annotation of genes associated to PTS-ptc encoding gene clusters was verified by BLAST searches. In addition, functional domains were identified using CD-Search [63].

### Phylogeny reconstruction

In order to obtain accurate phylogenies, the best fit models of amino acid substitution were selected using the program ProtTest [64]. The Akaike Information Criterion (AIC), which allows for a comparison of likelihoods from non-nested models, was adopted to select the best model [65] that was rtREV [28] for the three protein sets. The selected model was implemented in PHYML [66] to obtain maximum likelihood trees for the different alignments. Bootstrap support values were obtained from 1000 pseudo replicates.

MrBayes 3.1.2 [67] was used to obtain posterior probabilities of the nodes in the ML trees based on a Bayesian methodology. The program approximates the posterior probabilities of the phylogenetic trees using a Markov Chain Monte Carlo (MCMC) method. For the three alignments we used the same model used in PHYML in two replicates running four chains during a number of generations enough to ensure convergence of the sampled parameters. Convergence was verified using Tracer 1.4 [68] and accepted when the effective sample size of all the parameters was larger than 100, the recommended minimum effective size [68]. EI, HPr and HprK required 2.4 × 10^6^, 2.8 × 10^6 ^and 1 × 10^6 ^generations, respectively. Once convergence was assessed, the two runs were combined to obtain an estimate of the posterior distribution of the different parameters and the tree topology, with the first 10% samples discarded as burn-in.

For selected groups we carried out a comparison between the gene tree phylogeny and the 16S rRNA topology as a reference tree for the corresponding species. Shimodaira-Hasegawa's test [69] implemented in the program TreePuzzle 5.2 was used to determine whether the likelihood of the data associated to the two test trees was significantly different at an alpha level of 0.05 (a value above the threshold indicating a non-significant difference). Phylogenetic trees of sequences encoding 16S rRNA were obtained using the tools implemented in the Ribosomal Database Project II [70] and compared to maximum likelihood phylogenetic trees of the corresponding EI sequences obtained as indicated above. Since the Tree Builder tool of the Ribosomal Database Project has a limit of fifty sequences, in order to obtain a phylogenetic tree encompassing all species encoding a PTS-ptc, sequences of genes encoding 16S rRNA were aligned using ClustalW and their phylogenetic relationships were inferred using the PHYML maximum likelihood algorithm under the GTR model of nucleotide substitution and proportion of invariant and rate heterogeneity categories estimated from the data set. Support for the nodes in the resulting topologies was assessed by analyzing 500 bootstrap pseudo replicates.

Preliminary analyses suggested that some sequences might have experienced an accelerated rate of evolution with respect to other sequences in the alignment. In order to explore this possibility, Tajima's relative rate tests [71] were carried out.

## Abbreviations

AIC: Akaike Information Criterion; BPP: Bayesian posterior probability; BS: Bootstrap support; CCR: carbon catabolite repression; EI: Enzyme I; EI^Ntr^: nitrogen EI; FPr: Fructose-inducible HPr; GAF: domain present in c**G**MP-specific and -stimulated phosphodiesterases, *Anabaena ***a**denylate cyclases and *E. coli ***F**hlA; GTR: General Time Reversible model of nucleotide substitution; HGT: Horizontal Gene Transfer; HPr: Heat-stable, histidine-phosphorylatable protein; HPrK: HPr kinase; IIA^Fru^: fructose-class IIA protein; IIA^Glu^: glucose-class IIA protein; IIA^Man^: mannose-class IIA protein; IIA^Ntr^: fructose-class IIA protein involved in nitrogen metabolism; JTT: Jones-Taylor-Thornton model of amino acid substitution; MTPs: multiphosphoryl transfer proteins; NPr: nitrogen HPr; PTS: phosphoenolpyruvate:carbohydrate phosphotransferase system; PTS-ptc: PTS phosphoryl transfer chain; VPES: *Enterobacteriaceae*, *Pasteurellaceae*, *Shewanellaceae *and *Vibrionaceae*.

## Authors' contributions

IC carried out the molecular phylogenetic studies, participated in the sequence alignment and helped to draft the manuscript. FG–C participated in the design of the study, supervised the molecular phylogenetic studies and helped to draft the manuscript. MZ conceived of the study, carried out the database searches and genomic context analysis, participated in its design and coordination and drafted the manuscript. All authors read and approved the final manuscript.

## Supplementary Material

Additional file 1 Supplementary tables. Supplementary Table 1 lists the organisms included in this study. Supplementary Table 2 lists accession numbers and characteristics of the sequences utilized in this study. Click here for file

Additional file 2 Supplementary figures. Figure S1: likelihood mapping analysis. Figures S2 and S3: maximum likelihood phylogenetic trees for EI and HPr sequences, respectively. Figure S4: schematic representation of the proposed ancestral *rpoN *gene cluster of *Proteobacteria*. Figure S5: comparison of the topologies of phylogenetic trees for 16S and EI sequences belonging to groups R, Ntr and T (VPES). Figure S6: phylogenetic reconstruction of 16S rRNA sequences of the strains used in this study harbouring genes encoding EI, HPr or HPrK. Figure S7: *ptsH *or *ptsI *gene clusters of *Actinobacteria*. Figure S8: gene clusters containing FPr encoding genes and related homologues. Figure S9: sequence alignment of the IIA^Fru ^and the intervening domain of FPr proteins, *Acinetobacter *sp. *FruB *protein, and the tandem IIA^Fru ^domains of *Pseudomonas FruA *proteins. Figure S10: *ptsK *gene clusters of *Bacillales*. Figure S11: *ptsK *gene clusters of *Firmicutes *and *F. nucleatum*. Figure S12: *ptsH *or *ptsI *gene clusters of *Firmicutes*, VPES, *Borrelia *and *F. nucleatum*. Click here for file
