# Supplementary material for: Unraveling the evolutionary history of the phosphoryl-transfer chain of the phosphoenolpyruvate:phosphotransferase system through phylogenetic analyses and genome context
Source: BMC Evol Biol. 2008 May 16;8:147. doi: 10.1186/1471-2148-8-147 (PMC2405797; doi:10.1186/1471-2148-8-147)
Supplement: Additional file 1 — Supplementary tables. Supplementary Table 1 lists the organisms included in this study. Supplementary Table 2 lists accession numbers and characteristics of the sequences utilized in this study. [file 1471-2148-8-147-S1.pdf]

Supplementary Table 1. Organisms included in this study

	Taxonomic classification	Strains[a]	Nº species
Archaea			
Crenarchaeota	*Thermoprotei; Desulfurococcales; Desulfurococcaceae*	*Aeropyrum pernix* K1	1
	Sulfolobales; Sulfolobaceae	*Sulfolobus solfataricus* P2; *Sulfolobus tokodaii* str. 7	2
	Thermoproteales; Thermoproteaceae	*Pyrobaculum aerophilum* str. IM2	1
Euryarchaeota	*Archaeoglobi; Archaeoglobales; Archaeoglobaceae*	*Archaeoglobus fulgidus* DSM 4304	1
	Halobacteria; Halobacteriales; Halobacteriaceae	*Haloarcula marismortui* ATCC 43049; *Halobacterium* sp. NRC-1	2
	Methanobacteria; Methanobacteriales; Methanobacteriaceae	*Methanothermobacter thermautotrophicus* str. Delta H	1
	Methanococci; Methanococcales; Methanocaldococcaceae	*Methanocaldococcus jannaschii* DSM 2661	1
	Methanococcaceae	*Methanococcus maripaludis* S2	1
	Methanomicrobia; Methanosarcinales; Methanosarcinaceae	*Methanosarcina acetivorans* C2A; *Methanosarcina mazei* Go1	2
	Methanopyri; Methanopyrales; Methanopyraceae	*Methanopyrus kandleri* AV19	1
	Thermococci; Thermococcales; Thermococcaceae	*Pyrococcus abyssi* GE5; *Pyrococcus furiosus* DSM 3638; *Pyrococcus horikoshii* OT3; *Thermococcus kodakarensis* KOD1	4
	Thermoplasmata; Thermoplasmatales; Picrophilaceae	*Picrophilus torridus* DSM 9790	1
	Thermoplasmataceae	*Thermoplasma acidophilum* DSM 1728; *Thermoplasma volcanium* GSS1	2
Nanoarchaeota		*Nanoarchaeum equitans* Kin4-M	1
Bacteria			
Aquificae	*Aquificales; Aquificaceae*	*Aquifex aeolicus* VF5	1
Thermotogae	*Thermotogales; Thermotogaceae*	*Thermotoga maritima* MSB8	1
Deinococcus-	*Deinococci; Deinococcales;*	*Deinococcus radiodurans* R1	1

Thermus	*Deinococcaceae*		
	Thermales; Thermaceae	*Thermus thermophilus* HB27; *Thermus thermophilus* HB8	1
Cyanobacteria	*Chroococcales*	*Synechococcus elongatus* PCC 6301; *Synechococcus* sp. WH 8102; *Synechocystis* sp. PCC 6803; *Thermosynechococcus elongatus* BP-1	4
	Gloeobacteria; Gloeobacterales	*Gloeobacter violaceus* PCC 7421	1
	Nostocales; Nostocaceae	*Nostoc* sp. PCC 7120	1
	Prochlorales; Prochlorococcaceae	*Prochlorococcus marinus* str. MIT 9313; *Prochlorococcus marinus* subsp. *marinus* str. CCMP1375; *Prochlorococcus marinus* subsp. *pastoris* str. CCMP1986	1
Actinobacteria	*Actinobacteridae; Actinomycetales; Corynebacterineae; Corynebacteriaceae*	*Corynebacterium diphtheriae* NCTC 13129; *Corynebacterium efficiens* YS-314; *Corynebacterium glutamicum* ATCC 13032	3
	Mycobacteriaceae	*Mycobacterium avium* subsp. *paratuberculosis* str. k10; *Mycobacterium bovis* AF2122/97; *Mycobacterium leprae* TN; *Mycobacterium tuberculosis* CDC1551; *Mycobacterium tuberculosis* H37Rv	4
	Nocardiaceae	*Nocardia farcinica* IFM 10152	1
	Micrococcineae; Cellulomonadaceae	*Tropheryma whipplei* TW08/27; *Tropheryma whipplei* str. Twist	1
	Microbacteriaceae	*Leifsonia xyli* subsp. *xyli* str. CTCB07	1
	Propionibacterineae; Propionibacteriaceae	*Propionibacterium acnes* KPA171202	1
	Streptomycineae; Streptomycetaceae	*Streptomyces avermitilis* MA-4680; *Streptomyces coelicolor* A3(2)	2
	Bifidobacteriales ; Bifidobacteriaceae	*Bifidobacterium longum* NCC2705	1
	unclassified	*Symbiobacterium thermophilum* IAM 14863	1
Firmicutes	*Bacillales; Bacillaceae*	*Bacillus anthracis* str. 'Ames Ancestor'; *Bacillus anthracis* str. Sterne; *Bacillus anthracis* str. A2012; *Bacillus cereus* ATCC 10987; *Bacillus cereus* ATCC 14579; *Bacillus cereus* E33L; *Bacillus clausii* KSM-K16;	9

		Bacillus halodurans C-125; *Bacillus licheniformis* ATCC 14580; *Bacillus subtilis* subsp. *subtilis* str. 168; *Bacillus thuringiensis* serovar konkukian str. 97-27 ; *Geobacillus kaustophilus* HTA426; *Oceanobacillus iheyensis* HTE831	
	Listeriaceae	*Listeria innocua* Clip11262; *Listeria monocytogenes* EGD-e; *Listeria monocytogenes* str. 4b F2365	2
	Staphylococcaceae	*Staphylococcus aureus* subsp. *aureus* COL; *Staphylococcus aureus* subsp. *aureus* MRSA252; *Staphylococcus aureus* subsp. *aureus* MSSA476; *Staphylococcus aureus* subsp. *aureus* MW2; *Staphylococcus aureus* subsp. *aureus* Mu50; *Staphylococcus aureus* subsp. *aureus* N315; *Staphylococcus epidermidis* ATCC 12228; *Staphylococcus epidermidis* RP62A	2
	Lactobacillales; Enterococcaceae	*Enterococcus faecalis* V583	1
	Lactobacillaceae	*Lactobacillus acidophilus* NCFM; *Lactobacillus johnsonii* NCC 533; *Lactobacillus plantarum* WCFS1	3
	Streptococcaceae	*Lactococcus lactis* subsp. *lactis* Il1403; *Streptococcus agalactiae* 2603V/R; *Streptococcus agalactiae* NEM316; *Streptococcus mutans* UA159; *Streptococcus pneumoniae* R6; *Streptococcus pyogenes* M1 GAS; *Streptococcus pyogenes* MGAS10394; *Streptococcus pyogenes* MGAS315; *Streptococcus pyogenes* MGAS8232; *Streptococcus pyogenes* SSI-1; *Streptococcus thermophilus* CNRZ1066; *Streptococcus thermophilus* LMG 18311	6
	Clostridia; Clostridiales; Clostridiaceae	*Clostridium acetobutylicum* ATCC 824; *Clostridium perfringens* str. 13; *Clostridium tetani* E88	3
	Thermoanaerobacteriales;	*Thermoanaerobacter*	1

	Thermoanaerobacteriaceae	*tengcongensis* MB4	
	Mollicutes; Acholeplasmatales; Acholeplasmataceae	Onion yellows phytoplasma OY-M	1
	Entomoplasmatales; Entomoplasmataceae	*Mesoplasma florum* L1	1
	Mycoplasmatales; Mycoplasmataceae	*Mycoplasma gallisepticum* R; *Mycoplasma hyopneumoniae* 232; *Mycoplasma mobile* 163K; *Mycoplasma mycoides* subsp. *mycoides* SC str. PG1; *Mycoplasma penetrans* HF-2; *Mycoplasma pneumoniae* M129; *Mycoplasma pulmonis* UAB CTIP; *Ureaplasma parvum* serovar 3 str. ATCC 700970	8
Fusobacteria	*Fusobacterales; Fusobacteriaceae*	*Fusobacterium nucleatum* subsp. *nucleatum* ATCC 25586	1
Chloroflexi	*Dehalococcoidetes*	*Dehalococcoides ethenogenes* 195	1
Bacteroidetes-Chlorobi group	*Bacteroidetes ; Bacteroidales ; Bacteroidaceae*	*Bacteroides fragilis* YCH46; *Bacteroides fragilis* NCTC 9343; *Bacteroides thetaiotaomicron* VPI-5482	2
	Porphyromonadaceae	*Porphyromonas gingivalis* W83	1
	Chlorobi; Chlorobia; *Chlorobiales; Chlorobiaceae; Chlorobium/Pelodictyon* group	*Chlorobium tepidum* TLS	1
Planctomycetes	*Planctomycetacia; Planctomycetales; Planctomycetaceae*	*Rhodopirellula baltica* SH 1	1
Spirochaetes	*Spirochaetales; Leptospiraceae*	*Leptospira interrogans* serovar Copenhageni str. Fiocruz L1-130; *Leptospira interrogans* serovar Lai str. 56601	1
	Spirochaetaceae	*Borrelia burgdorferi* B31; *Borrelia garinii* PBi; *Treponema denticola* ATCC 35405; *Treponema pallidum* subsp. *pallidum* str. Nichols	4
Chlamydiae-Verrucomicrobia	*Chlamydiae ; Chlamydiales ; Chlamydiaceae*	*Chlamydia muridarum* Nigg; *Chlamydia trachomatis* D/UW-3/CX; *Chlamydophila abortus* S26/3; *Chlamydophila caviae* GPIC; *Chlamydophila pneumoniae* AR39; *Chlamydophila pneumoniae* CWL029; *Chlamydophila*	5

		pneumoniae J138; *Chlamydophila pneumoniae* TW-183	
	Parachlamydiaceae	*Candidatus Protochlamydia amoebophila* UWE25	1
Proteobacteria	*Alphaproteobacteria; Caulobacterales; Caulobacteraceae*	*Caulobacter crescentus* CB15	1
	Rhizobiales; Bartonellaceae	*Bartonella henselae* str. Houston-1; *Bartonella quintana* str. Toulouse	2
	Bradyrhizobiaceae	*Bradyrhizobium japonicum* USDA 110; *Rhodopseudomonas palustris* CGA009	2
	Brucellaceae	*Brucella abortus* biovar 1 str. 9-941; *Brucella melitensis* 16M; *Brucella suis* 1330	3
	Phyllobacteriaceae	*Mesorhizobium loti* MAFF303099	1
	Rhizobiaceae	*Agrobacterium tumefaciens* str. C58; *Sinorhizobium meliloti* 1021	2
	Rhodobacterales; Rhodobacteraceae	*Silicibacter pomeroyi* DSS-3	1
	Rhodospirillales; Acetobacteraceae	*Gluconobacter oxydans* 621H	1
	Rickettsiales; Anaplasmataceae	*Anaplasma marginale* str. St. Maries; *Ehrlichia ruminantium* str. Gardel; *Ehrlichia ruminantium* str. Welgevonden	2
	Rickettsiaceae	*Rickettsia conorii* str. Malish 7; *Rickettsia prowazekii* str. Madrid E; *Rickettsia typhi* str. Wilmington; *Wolbachia* endosymbiont of *Drosophila melanogaster*; *Wolbachia* endosymbiont strain TRS of *Brugia malayi*	5
	Sphingomonadales; Sphingomonadaceae	*Zymomonas mobilis* subsp. *mobilis* ZM4	1
	Betaproteobacteria; Burkholderiales; Alcaligenaceae	*Bordetella bronchiseptica* RB50; *Bordetella parapertussis* 12822; *Bordetella pertussis* Tohama I	3
	Burkholderiaceae	*Burkholderia mallei* ATCC 23344; *Burkholderia pseudomallei* K96243; *Ralstonia solanacearum* GMI1000	3
	Neisseriales; Neisseriaceae	*Chromobacterium violaceum* ATCC 12472; *Neisseria gonorrhoeae* FA 1090; *Neisseria meningitidis* MC58; *Neisseria meningitidis* Z2491	3

	Nitrosomonadales; Nitrosomonadaceae	*Nitrosomonas europaea* ATCC 19718	1
	Deltaproteobacteria; Bdellovibrionales; Bdellovibrionaceae	*Bdellovibrio bacteriovorus* HD100	1
	Desulfobacterales ; Desulfobulbaceae	*Desulfotalea psychrophila* LSv54	1
	Desulfovibrionales; Desulfovibrionaceae	*Desulfovibrio vulgaris* subsp. *vulgaris* str. Hildenborough	1
	Desulfuromonadales; Geobacteraceae	*Geobacter sulfurreducens* PCA	1
	Epsilonproteobacteria; Campylobacterales; Campylobacteraceae	*Campylobacter jejuni* RM1221; *Campylobacter jejuni* subsp. *jejuni* NCTC 11168	1
	Helicobacteraceae	*Helicobacter hepaticus* ATCC 51449; *Helicobacter pylori* 26695; *Helicobacter pylori* J99; *Wolinella succinogenes* DSM 1740	3
	Proteobacteria; Gammaproteobacteria; Alteromonadales; Idiomarinaceae	*Idiomarina loihiensis* L2TR	1
	Shewanellaceae	*Shewanella oneidensis* MR-1	1
	Enterobacteriales; Enterobacteriaceae	*Buchnera aphidicola* str. APS; *Buchnera aphidicola* str. Bp; *Buchnera aphidicola* str. Sg; Candidatus *Blochmannia floridanus*; *Erwinia carotovora* subsp. *atroseptica* SCRI1043; *Escherichia coli* CFT073; *Escherichia coli* K12; *Escherichia coli* O157:H7; *Escherichia coli* O157:H7 EDL933; *Photorhabdus luminescens* subsp. *laumondii* TTO1; *Salmonella enterica* subsp. *enterica* serovar Choleraesuis str. SC-B67; *Salmonella enterica* subsp. *enterica* serovar Paratyphi A str. ATCC 9150; *Salmonella enterica* subsp. *enterica* serovar Typhi Ty2; *Salmonella enterica* subsp. *enterica* serovar Typhi str. CT18; *Salmonella typhimurium* LT2; *Shigella flexneri* 2a str. 2457T; *Shigella flexneri* 2a str. 301; *Wigglesworthia glossinidia*; *Yersinia pestis* CO92; *Yersinia pestis* KIM; *Yersinia pestis* biovar Medievalis str. 91001; *Yersinia*	10

		pseudotuberculosis IP 32953	
	Legionellales; Coxiellaceae	*Coxiella burnetii* RSA 493	1
	Legionellaceae	*Legionella pneumophila* str. Lens; *Legionella pneumophila* str. Paris; *Legionella pneumophila* subsp. *pneumophila* str. Philadelphia 1;	1
	Methylococcales; Methylococcaceae	*Methylococcus capsulatus* str. Bath	1
	Pasteurellales; Pasteurellaceae	*Haemophilus ducreyi* 35000HP; *Haemophilus influenzae* Rd KW20; *Mannheimia succiniciproducens* MBEL55E; *Pasteurella multocida* subsp. *multocida* str. Pm70	4
	Pseudomonadales; Moraxellaceae	*Acinetobacter* sp. ADP1	1
	Pseudomonadaceae	*Pseudomonas aeruginosa* PAO1; *Pseudomonas putida* KT2440; *Pseudomonas syringae* pv. tomato str. DC3000	3
	Thiotrichales; Francisellaceae	*Francisella tularensis* subsp. *tularensis* SCHU S4	1
	Vibrionales; Vibrionaceae	*Photobacterium profundum* SS9; *Vibrio cholerae* O1 biovar eltor str. N16961; *Vibrio fischeri* ES114; *Vibrio parahaemolyticus* RIMD 2210633; *Vibrio vulnificus* CMCP6; *Vibrio vulnificus* YJ016	5
	Xanthomonadales; Xanthomonadaceae	*Xanthomonas axonopodis* pv. citri str. 306; *Xanthomonas campestris* pv. *campestris* str. ATCC 33913; *Xanthomonas oryzae* pv. *oryzae* KACC10331; *Xylella fastidiosa* 9a5c; *Xylella fastidiosa* Temecula1	4

[a] Strains harbouring PTS genes are indicated in red.

Table 2. Sequences used in this study.

Acc. N°	Organism[a]	Taxonomic classification	Aliases[b]	Domains	Group[c]	Cluster[d]	Observations
YP_020909	*Bacillus anthracis*	*Firmicutes; Bacilli; Bacillales; Bacillaceae*	banthrax	EI	T2	10	
YP_020910	str. “Ames”		Bcere14	HPr	T2	10	
YP_022040	Ancestor[e]		Bcerecrh	HPr		11	Crh
YP_022051			Banthrhk	HPrK		11	
YP_176154	*Bacillus clausii*		bclausii	EI	T2	10	
YP_176155	KSM-K16		bclaus	HPr	T2	10	
YP_176734			bclaushx	HPr		11	
YP_176528			bclaucrh	HPr		11	Crh
YP_176550			bclaushk	HPrK		11	
NP_980409	*Bacillus cereus*		Bcereus10	EI	T2	10	
NP_980410	ATCC 10987		Bcere10	HPr	T2	10	
NP_981549			Bcerecrh	HPr		11	Crh
NP_981560			Bcer10hk	HPrK		11	
NP_833766	*Bacillus cereus*		Bcereus14	EI	T2	10	
NP_833767	ATCC 14579		Bcere14	HPr	T2	10	
NP_834817			Bcerecrh	HPr		11	Crh
NP_834828			Bcer14hk	HPrK		11	
YP_085385	*Bacillus cereus* ZK		bcereuszk	EI	T2	10	
YP_085386	E33L		Bcere14	HPr	T2	10	
YP_086416			Bcerecrh	HPr		11	Crh
YP_086427			Banthrhk	HPrK		11	
NP_243939	*Bacillus halodurans*		bhalodu	EI	T2	10	
NP_243940	C-125		bhalo	HPr	T2	10	
NP_244433			bhalocrh	HPr		11	Crh
NP_244457			bhalohk	HPrK		11	
YP_091192	*Bacillus*		blichen	EI	T2	10	
YP_091191	*licheniformis*		blich	HPr	T2	10	
YP_093234	ATCC 14580		Blichcrh1	HPr		11	Crh
YP_091681			Blichcrh2	HPr		11	Crh
YP_093257			blichehk	HPrK		11	
NP_389274	*Bacillus subtilis*		bsubtil	EI	T2	10	
NP_389273			bsubti	HPr	T2	10	
NP_391354			bsubticrh	HPr		11	Crh
NP_391380			bsubtihk	HPrK		11	
YP_038108	*Bacillus*		bthuring	EI	T2	10	
YP_038109	*thuringiensis*		Bcere14	HPr	T2	10	
YP_039139	serovar konkukian		Bcerecrh	HPr		11	Crh
YP_039150	str. 97-27		Banthrhk	HPrK		11	
YP_146849	*Geobacillus kaustophilus*		gbkaust	EI	T2	10	
YP_146848	HTA426		gkaust	HPr	T2	10	
YP_148916			gkaustcrh	HPr		11	Crh
YP_148935			gkausthk	HPrK		11	
NP_693353	*Oceanobacillus*		oihey	EI	T2	10	

NP_693265	*iheyensis*		oihey	HPr	T2	10
NP_693386			oiheycrh	HPr	Crh	11
NP_693403			Oiheyhk1	HPrK		11
NP_694350			Oiheyhk2	HPrK		11
NP_470339	*Listeria innocua*	*Firmicutes; Bacilli; Bacillales; Listeriaceae*	linnoc	EI	T2	10
NP_470338	Clip11262		Lmono	HPr	T2	10
NP_471956			linnohk	HPrK		
NP_464528	*Listeria*		lmonoegde	EI	T2	10
NP_464527	*monocytogenes*		Lmono	HPr	T2	10
NP_466006	EGD-e[f]		lmonohk	HPrK		11
YP_043143	*Staphylococcus*	*Firmicutes; Bacilli; Bacillales; Staphylococcaceae*	Saurmw2	EI	T2	10
YP_043142	*aureus* subsp. *aureus*		Saure	HPr	T2	10
YP_042853	MSSA476		Saurehk	HPrK		11
YP_185956	*Staphylococcus*		saurcol	EI	T2	10
YP_185955	*aureus* subsp. *aureus*		Saure	HPr	T2	10
YP_185699	COL		Saurehk	HPrK		11
YP_040471	*Staphylococcus*		saurmrsa	EI	T2	10
YP_040470	*aureus* subsp. *aureus*		Saure	HPr	T2	10
YP_040241	MRSA252		Saurehk	HPrK		11
NP_374203	*Staphylococcus*		Saurne315	EI	T2	10
NP_374202	*aureus* subsp. *aureus*		Saure	HPr	T2	10
NP_373970	N315		Saurehk	HPrK		11
NP_371608	*Staphylococcus*		Saurmu50	EI	T2	10
NP_371607	*aureus* subsp. *aureus*		Saure	HPr	T2	10
NP_371284	Mu50		Saurehk	HPrK		11
NP_764337	*Staphylococcus*		sepiderm	EI	T2	10
NP_764336	*epidermidis* ATCC		sepider	HPr	T2	10
NP_764098	12228[g]		sepiderhk	HPrK		11
NP_814461	*Enterococcus*	*Firmicutes; Bacilli; Lactobacillales; Enterococcaceae*	efaecal	EI	T2	10
NP_814460	*faecalis* V583		efaec	HPr	T2	10
NP_815452			efaechk	HPrK		12
YP_193550	*Lactobacillus*	*Firmicutes; Bacilli; Lactobacillales; Lactobacillaceae*	lbacido	EI	T2	10
YP_193549	*acidophilus* NCFM		lbacid	HPr	T2	10
YP_193583			lbacidohk	HPrK		12
NP_964672	*Lactobacillus*		lbjohn	EI	T2	10
NP_964671	*johnsonii* NCC 533		lbjohn	HPr	T2	10
NP_964704			lbjohnhk	HPrK		12
NP_784928	*Lactobacillus plantarum* WCFS1		Lbplant	EI	T2	10
NP_784927			lbplan	HPr	T2	10
NP_784505			lbplanthk	HPrK		12
NP_266274	*Lactococcus lactis*	*Firmicutes; Bacilli; Lactobacillales; Streptococcaceae*	Llactis	EI	T2	10
NP_266273	Il1403		llacti	HPr	T2	10
NP_266774			llacthk	HPrK		
NP_687837	*Streptococcus*		Stagal26	EI	T2	10
NP_687836	*agalactiae* 2603V/R		Sagal	HPr	T2	10
NP_687751			Sagalhk	HPrK		12

NP_735290	*Streptococcus*		stagalnem	EI	T2	10	
NP_735289	*agalactiae* NEM316		Sagal	HPr	T2	10	
NP_735207			Sagalhk	HPrK		12	
NP_721103	*Streptococcus*		Stmutans	EI	T2	10	
NP_721102	*mutans* UA159		smuta	HPr	T2	10	
NP_721171			smutanhk	HPrK		12	
NP_358656	*Streptococcus*		stpneumon	EI	T2	10	
NP_358657	*pneumoniae* R6[h]		spneu	HPr	T2	10	
NP_358863			Spneur6hk	HPrK		12	
NP_664850	*Streptococcus pyogenes* MGAS315		Spyog315	EI	T2	10	Start codon corrected to 1089433 in NC_004070
NP_664851			Spyog	HPr	T2	10	
NP_664216			Spyo15hk	HPrK		12	
NP_607480	*Streptococcus*		Stpyog	EI	T2	10	
NP_607481	*pyogenes*		Spyog	HPr	T2	10	
NP_606834	MGAS8232[i]		Spyo32hk	HPrK		12	
YP_139711	*Streptococcus*		sthermlm	EI	T2	10	
YP_139712	*thermophilus* LMG		Stherml	HPr	T2	10	
YP_139186	18311		sthermlhk	HPrK		12	
YP_141623	*Streptococcus*		Stthermcn	EI	T2	10	
YP_141624	*thermophilus*		sthermc	HPr	T2	10	
YP_141077	CNRZ1066		sthermchk	HPrK		12	
AAK81027	*Clostridium*	*Firmicutes; Clostridia; Clostridiales; Clostridiaceae*	Cacetobut	EI	T2	10	
NP_348445	*acetobutylicum*		cacet	HPr	T2	10	
NP_349688	ATCC 824		cacetrocr	HPr/RocR		10	
NP_347723			cacethk	HPrK		12	
NP_563273	*Clostridium*		Cperfrin	EI	T2	10	
NP_562585	*perfringens* str. 13		cperf	HPr	T2	10	
NP_563274			cperfrocr	HPr/RocR		10	
NP_561920			cperfhk	HPrK		12	
NP_782363	*Clostridium tetani*		ctetani	EI	T2	10	
NC_004557	E88		cteth	HPr	T2	10	Not annotated; coding sequence 1390790-1391044
NP_781659			ctethk	HPrK		12	
NP_623871	*Thermoanaerobacter*	*Firmicutes; Clostridia; Thermoanaerobacteriales;*	ttencong	EI	T2	10	
NP_623408	*tengcongensis* MB4	*Thermoanaerobacteriaceae*	tteng	HPr		10	
NP_623539			ttenghk	HPrK		12	
YP_053761	*Mesoplasma florum*	*Firmicutes; Mollicutes; Entomoplasmatales;*	meflorum	EI	T2	10	
YP_053807	L1	*Entomoplasmataceae*		HPr	T2	10	
YP_053302			meflorhk	HPrK		12	
NP_852862	*Mycoplasma*	*Firmicutes; Mollicutes; Mycoplasmatales;*	mygalli	EI	T2	10	
NP_852811	*gallisepticum* R	*Mycoplasmataceae*	mgalli	HPr	T2	10	
NP_853498			mygalhk	HPrK		12	
NP_073100	*Mycoplasma*		mygenit	EI	T2	10	
NP_072701	*genitalium* G-37		mgenith	HPr	T2	10	
NP_072747			mygenhk	HPrK		12	
YP_115980	*Mycoplasma*		mypneum	EI	T2	10	

YP_116136	*hyopneumoniae* 232		mhyopn	HPr	T2	10	
YP_015758	*Mycoplasma mobile*		mymobil	EI	T2	10	
YP_016103	163K		mmobh	HPr	T2	10	
YP_015879			mymobhk	HPrK		12	
NP_975272	*Mycoplasma*		mymycoid	EI	T2	10	
NP_975744	*mycoides* subsp.		mmyco	HPr	T2	10	
NP_975907	*mycoides* SC		mmychk	HPrK		12	
NP_757931	*Mycoplasma*		mypenet	EI	T2	10	
NP_757668	*penetrans* HF-2		mpenet	HPr	T2	10	
NP_757687			mypenhk	HPrK		12	
NP_110316	*Mycoplasma*		Mypnem129	EI	T2	10	
NP_109741	*pneumoniae* M129		mpneu	HPr	T2	10	
NP_109911			mypnehk	HPrK		12	
NP_326433	*Mycoplasma*		mypulmon	EI	T2	10	
NP_326434	*pulmonis* UAB CTIP		mpulmoh	HPr	T2	10	
NP_326542			mypulhk	HPrK		12	
NP_078426	*Ureaplasma parvum*		uparv	HPr	T2	10	
NP_077906	serovar 3 str. ATCC 700970		uparvhk	HPrK		12	
NP_419666	*Caulobacter*	*Proteobacteria; Alphaproteobacteria;;*	ccrescgaf	GAF/EI	Ntr		
NP_419060	*crescentus* CB15	*Caulobacterales*	ccresc	HPr	R/Ntr	8	NPr
NP_419356		*Caulobacteraceae*	Ccreaghe1	IIA^{Glu}/HPr/EI	T1		
NP_419267			Ccreaghe2	IIA^{Glu}/HPr/EI	T1		
NP_419058			ccreschk	HPrK		8	
YP_032997	*Bartonella henselae*	*Proteobacteria; Alphaproteobacteria; Rhizobiales;*	bhenafhe	IIA^{Fru}/HPr/EI	T1	14	
YP_032923	str. Houston-1	*Bartonellaceae*	bhenseh	HPr	R/Ntr	8	NPr; Start codon corrected to 81811 in NC_005956
YP_032925			bhenshk	HPrK		8	
YP_031850	*Bartonella quintana*		bquiafhe	IIA^{Fru}/HPr/EI	T1	14	
YP_031778	str. Toulouse		bquinh	HPr	R/Ntr	8	NPr
YP_031780			bquinhk	HPrK		8	
NP_770215	*Bradyrhizobium*	*Proteobacteria; Alphaproteobacteria; Rhizobiales;*	bjapo	EI	T1	16	
NP_774788	*japonicum* USDA	*Bradyrhizobiaceae*	Bjapoh2	HPr	R/Ntr	8	NPr
NP_770214	110		Bjapoh1	HPr		16	
NP_766857			bajapogaf	GAF/EI	Ntr		
NP_774786			bjapohk	HPrK		8	
NP_945958	*Rhodopseudomonas*		rpalusgaf	GAF/EI	Ntr		
NP_945707	*palustris* CGA009		rpalus	HPr	R/Ntr	8	NPr
NP_945709			rpalushk	HPrK		8	
YP_222524	*Brucella abortus* biovar	*Proteobacteria; Alphaproteobacteria; Rhizobiales;*	babortgaf	GAF/EI	Ntr		
YP_222731	1 str. 9-941	*Brucellaceae*	Baborth	HPr	R/Ntr	8	NPr
YP_222728			baborhk	HPrK		8	
NP_539108	*Brucella melitensis* 16M		bmelitgaf	GAF/EI	Ntr		
NP_540948			bmelith	HPr	R/Ntr	8	NPr
NP_540951			baborhk	HPrK		8	
NP_698850	*Brucella suis* 1330		bsuisgaf	GAF/EI	Ntr		
NP_699070			Baborth	HPr	R/Ntr	8	NPr

NP_699067			baborhk	HPrK		8	
NP_104682	*Mesorhizobium loti* MAFF303099	*Proteobacteria; Alphaproteobacteria; Rhizobiales; Phyllobacteriaceae*	Mloti1 Mlotih3	HPr/EI	T1	16	Start codon moved to pos. 2913056 in NC_002678; coding sequence 2911074-2913056 (complement)
NP_105981			Mloti2	EI	T1	16	
NP_105813			Mlotih1	HPr	R/Ntr	8	NPr
NP_105982			Mlotih2	HPr		16	
NP_104538			mlotigaf	GAF/EI	Ntr		
NP_105815			mlotihk	HPrK		8	
NP_534657	*Agrobacterium*	*Proteobacteria; Alphaproteobacteria; Rhizobiales;*	atumegaf	GAF/EI	Ntr		
NP_530745	*tumefaciens* str. C58	*Rhizobiaceae; Rhizobium/Agrobacterium* group	atumeh	HPr	R/Ntr	8	NPr
NP_530747			atumehk	HPrK		8	
NP_386718	*Sinorhizobium*	*Proteobacteria; Alphaproteobacteria; Rhizobiales;*	smelitgaf	GAF/EI	Ntr		
NP_384146	*meliloti* 1021	*Rhizobiaceae; Sinorhizobium/Ensifer* group	smelith	HPr	R/Ntr	8	NPr
NP_384148			smelihk	HPrK		8	
YP_168238	*Silicibacter*	*Proteobacteria; Alphaproteobacteria; Rhodobacterales;*	spomergaf	GAF/EI	Ntr		
YP_165969	*pomeroyi* DSS-3	*Rhodobacteraceae*	spomerh	HPr	R/Ntr	8	NPr
YP_165966			spomerhk	HPrK		8	
YP_191242	*Gluconobacter*	*Proteobacteria; Alphaproteobacteria; Rhodospirillales;*	goxyda	EI	R	8	
YP_191243	*oxydans* 621H	*Acetobacteraceae*	goxydah	HPr	R/Ntr	8	
YP_191246			goxydahk	HPrK		8	
YP_163386	*Zymomonas mobilis*	*Proteobacteria; Alphaproteobacteria; e*	zmobigaf	GAF/EI	Ntr		
YP_163062	subsp. *mobilis* ZM4	*Sphingomonadales; Sphingomonadacea*	zmobih	HPr	R/Ntr	8	NPr
YP_163059			zmobihk	HPrK		8	
NP_884228	*Bordetella*	*Proteobacteria; Betaproteobacteria; Burkholderiales;*	bparapert	EI	R	8	
NP_884227	*parapertussis* 12822	*Alcaligenaceae*	Bparaph	HPr	R/Ntr	8	
NP_886180			Bbronhk	HPrK		8	
NP_880238	*Bordetella pertussis*		bpertus	EI	R	8	
NP_880237	Tohama I		Bparaph	HPr	R/Ntr	8	
NP_879527			bperthk	HPrK		8	
NP_888698	*Bordetella*		bbronchi	EI	R	8	
NP_888697	*bronchiseptica*		Bparaph	HPr	R/Ntr	8	
NP_891042	RB50		Bbronhk	HPrK		8	
YP_104687	*Burkholderia mallei*	*Proteobacteria; Betaproteobacteria; Burkholderiales;*	bumallei	EI	R	8	
YP_104688	ATCC 23344	*Burkholderiaceae*	Bmalleih	HPr	R/Ntr	8	
YP_104650			bmalaghe	IIA^{Glu}/HPr/EI	T1		
YP_104596			Bmallhk	HPrK		8	
YP_107065	*Burkholderia*		bupseud	EI	R	8	
YP_107064	*pseudomallei*		Bmalleih	HPr	R/Ntr	8	
YP_107123	K96243		bpseaghe	IIA^{Glu}/HPr/EI	T1		
YP_107155			Bmallhk	HPrK		8	
NP_518469	*Ralstonia*		rsolan	EI	R	8	
NP_518468	*solanacearum*		rsolanh	HPr	R/Ntr	8	
NP_522841	GMI1000		rsolaghe	IIA^{Glu}/HPr/EI	T1		Located in megaplasmid GMI1000
NP_520982			rsolafhe	IIA^{Fru}/HPr/EI	T1	14	

NP_518526			rsolanhk	HPrK		8	
NP_900486	*Chromobacterium*	*Proteobacteria; Betaproteobacteria; Neisseriales;*	chviola	EI	R	8	
NP_900485	*violaceum* ATCC	*Neisseriaceae*	chviolh	HPr	R/Ntr	8	
NP_901981	12472		chvioehaf	HPr/EI/IIA^{Fru}	T1	14	
NP_900650			Chviaghe1	IIA^{Glu}/HPr/EI	T1		
NP_900228			Chviaghe2	IIA^{Glu}/HPr/EI	T1		
NP_902722			Chviafhe	IIA^{Fru}/HPr/EI	T1	14	
NP_903005			cviolhk	HPrK		8	Start codon moved to 3632483 in NC_005085
NP_275034	*Neisseria*		nmeningmc	EI	R	8	
NP_275035	*meningitidis* MC58		Nmeningh	HPr	R/Ntr	8	
NP_273779			nmenimhk	HPrK		8	
NP_283222	*Neisseria*		Nmeningz2	EI	R	8	
NP_283221	*meningitidis* Z2491		Nmeningh	HPr	R/Ntr	8	
NP_283727			nmenizhk	HPrK		8	
YP_209058	*Neisseria*		ngonorr	EI	R	8	
YP_209057	*gonorrhoeae* FA		ngonorh	HPr	R/Ntr	8	
YP_207473	1090		ngonohk	HPrK		8	
NP_842189	*Nitrosomonas*	*Proteobacteria; Betaproteobacteria;*	niteurop	EI	R	8	
NP_842188	*europaea* ATCC	*Nitrosomonadales;*	neurop	HPr	R/Ntr	8	
NP_840160	19718	*Nitrosomonadaceae*	neurohk	HPrK		8	
YP_158597	*Azoarcus* sp. EbN1	*Proteobacteria; Betaproteobacteria; Rhodocyclales;*	azoarcus	EI	R	8	
YP_158596		*Rhodocyclaceae*	azoarch	HPr	R/Ntr	8	
YP_158941			azoarhk	HPrK		8	
YP_010050	*Desulfovibrio vulgaris*	*Proteobacteria*; delta/epsilon subdivisions;	desulfvul	EI	R	8	
YP_010051	subsp. *vulgaris* str.	*Deltaproteobacteria; Desulfovibrionales;*	dvulga	HPr	R/Ntr	8	
YP_010202	Hildenborough	*Desulfovibrionaceae*	dvulamhe	IIA^{Man}/HPr/EI		16	
NP_952930	*Geobacter*	*Proteobacteria*; delta/epsilon subdivisions;	Gsulfur1	EI	R	8	
NP_952218	*sulfurreducens* PCA	*Deltaproteobacteria; Desulfuromonadales; Geobacteraceae*	Gsulfur2	GAF/EI	Ntr		Start codon moved to 1271776 in NC_002939
NP_952931			gsulfurh	HPr	R/Ntr	8	
NP_952934			gsulfhk	HPrK		8	
YP_154896	*Idiomarina loihiensis*	*Proteobacteria; Gammaproteobacteria;*	iloihgaf	GAF/EI	Ntr		
YP_154784	L2TR	*Alteromonadales; Idiomarinaceae*	iloih	HPr	R/Ntr	8	NPr
NP_717833	*Shewanella oneidensis*	*Proteobacteria; Gammaproteobacteria;*	soneid	EI		10	
NP_717834	MR-1	*Alteromonadales; Shewanellaceae*	Soneidh1	HPr	T2	10	
NP_716952			soneidgaf	GAF/EI	Ntr		
NP_719495			Soneidh2	HPr	R/Ntr	8	NPr
NP_878790	Candidatus	*Proteobacteria; Gammaproteobacteria;*	bflorid	EI	T2	10	
NP_878789	*Blochmannia floridanus*	*Enterobacteriales; Enterobacteriaceae*	bfloridh	HPr	T2	10	
NP_239901	*Buchnera aphidicola* str.		baphiaps	EI	T2	10	
NP_239902	APS		baphiapsh	HPr	T2	10	
NP_660421	*Buchnera aphidicola* str.		baphisg	EI	T2	10	
NP_660422	Sg		baphisgh	HPr	T2	10	
NP_777694	*Buchnera aphidicola* str.		baphibp	EI	T2	10	
NP_777695	Bp		baphibph	HPr	T2	10	
YP_049001	*Erwinia carotovora*		ecaroto	EI	T2	10	

YP_049002	subsp. *atroseptica*	ecarotoh	HPr	T2	10	
YP_048411	SCRI1043	ecarothn	HPr	R/Ntr	8	NPr
YP_049093		ecarotgaf	GAF/EI	Ntr		
YP_050820		ecarafh	IIA^{Fru}/HPr		14	FPr
NP_754833	*Escherichia coli* CFT073	ecolicft	EI	T2	10	Deleted G at pos. 2814986; coding sequence 2814958-2816686
NP_754832		Ecolik12h	HPr	T2	10	
NP_756145		Ecolicft2	HPr			
NP_755832		Ecolkhn	HPr	R/Ntr	8	NPr
NP_755299		ecolcgaf	GAF/EI	Ntr		
NP_754804		ecolcheag	HPr/EI/IIA^{Fru}	T1	14	
NP_756760		ecolcheaf	HPr/EI/IIA^{Fru}	T1	14	
NP_754590		ecolcafh	IIA^{Fru}/HPr		14	FPr
NP_753561		ecolcygcg	IIA^{Man}/HPr/EI	T1	16	EI lacks C-t domain
NP_416911	*Escherichia coli* K12	Ecolik12	EI	T2	10	
NP_416910		Ecolik12h	HPr	T2	10	
NP_417673		Ecolkhn	HPr	R/Ntr	8	NPr
NP_417306		ecolkgaf	GAF/EI	Ntr		
NP_416884		ecolkheag	HPr/EI/IIA^{Fru}	T1	14	
YP_026278		ecolkheaf	HPr/EI/IIA^{Fru}	T1	14	
NP_416674		Ecolkafh[28]	IIA^{Fru}/HPr		14	FPr
NP_415716		ecolkycgc	IIA^{Man}/HPr/EI		16	EI lacks C-t domain
NP_311315	*Escherichia coli* O157:H7	Ecolio157	EI	T2	10	
NP_311314		Ecolik12h	HPr	T2	10	
NP_312381		Ecolio2	HPr			
NP_312112		Ecolkhn	HPr	R/Ntr	8	NPr
NP_311713		Ecologaf	GAF/EI	Ntr		
NP_311290		Ecoloheag	HPr/EI/IIA^{Fru}	T2	14	
NP_312903		ecoloheaf	HPr/EI/IIA^{Fru}	T2	14	
NP_311088		Ecolkafh	IIA^{Fru}/HPr		14	FPr
NP_309730		Ecoloycgc	IIA^{Man}/HPr/EI		16	EI lacks C-t domain
NP_288978	*Escherichia coli* O157:H7 EDL933	Ecolio157	EI	T2	10	
NP_288977		Ecolik12h	HPr	T2	10	
NP_290053		Ecolioe2	HPr			
NP_289780		Ecolkhn	HPr	R/Ntr	8	NPr
NP_289381		Ecologaf	GAF/EI	Ntr		
NP_288953		Ecoloheag	HPr/EI/IIA^{Fru}	T1	14	
NP_290584		ecoleheaf	HPr/EI/IIA^{Fru}	T1	14	Startcodon moved to 5017869 in NC_002655.2
NP_288752		ecooeafh	IIA^{Fru}/HPr		14	FPr
NP_287445		Ecoloycgc	IIA^{Man}/HPr/EI		16	EI lacks C-t domain
NP_928693	*Photorhabdus luminescens* subsp. *laumondii* TTO1	pluminis	EI	T2	10	
NP_928694		pluminish	HPr	T2	10	
NP_931242		plumihn	HPr	R/Ntr	8	NPr
NP_927966		plumingaf	GAF/EI	Ntr		
NP_929255		plumiafh	IIA^{Fru}/HPr		14	FPr
NC_003198	*Salmonella enterica* subsp. *enterica* serovar	sentt	EI	T2	10	Deleted T at pos. 2506765 in NC_003198; coding sequence

	Choleraesuis str. SC-B67					2505709-2507437
YP_217416		Ecolik12h	HPr	T2	10	
YP_218249		sentchn	HPr	R/Ntr	8	NPr
YP_217929		sentcgaf	GAF/EI	Ntr		
YP_218989		sentheaf	HPr/EI/IIA^{Fru}	T1	14	
YP_217209		Stypafh	IIA^{Fru}/HPr		14	FPr
YP_149756	*Salmonella enterica*	Senter	EI	T2	10	
YP_149757	subsp. *enterica* serovar	Ecolik12h	HPr	T2	10	
YP_152328	Paratyphi A str. ATCC	Styphhn	HPr	R/Ntr	8	NPr
YP_152025	9150	sentpgaf	GAF/EI	Ntr		
YP_149957		sentpafh	IIA^{Fru}/HPr		14	FPr
NP_804294	*Salmonella enterica*	Senter	EI	T2	10	
NP_804295	subsp. *enterica* serovar	Ecolik12h	HPr	T2	10	
NP_806917	Typhi Ty2	Styphhn	HPr	R/Ntr	8	NPr
NP_806606		senttgaf	GAF/EI	Ntr		
NP_804501		Stypafh	IIA^{Fru}/HPr		14	FPr
NP_461367	*Salmonella typhimurium*	Senter	EI	T2	10	
NP_461366	LT2	Ecolik12h	HPr	T2	10	
NP_462234		Styphhn	HPr	R/Ntr	8	NPr
NP_461920		sthyphgaf	GAF/EI	Ntr		
NP_462991		sentlheaf	HPr/EI/IIA^{Fru}	T1	14	
NP_461151		Stypafh	IIA^{Fru}/HPr		14	FPr
NP_708271	*Shigella flexneri* 2a str.	Ecolio157	EI	T2	10	
NP_708270	301	Ecolik12h	HPr	T2	10	
NP_709005		Ecolkhn	HPr	R/Ntr	8	NPr
NP_708618		Shflexgaf	GAF/EI	Ntr		
NP_708251		Shflex3heag	HPr/EI/IIA^{Fru}	T1	14	
NC_004337		Sfle3heaf	HPr/EI/IIA^{Fru}	T1	14	G replaced by A at pos. 4156230;<br>start codon moved to 4157288
NP_708066		Sflexafh	IIA^{Fru}/HPr		14	FPr
NP_707107		Sfleycgc	IIA^{Man}/HPr/EI		16	EI lacks C-t domain
NP_837981	*Shigella flexneri* 2a str.	Ecolio157	EI	T2	10	
NP_837980	2457T	Ecolik12h	HPr	T2	10	
NP_838715		Ecolkhn	HPr	R/Ntr	8	NPr
NP_838341		Shflexgaf	GAF/EI	Ntr		
NP_837959		Shflex2heag	HPr/EI/IIA^{Fru}	T1	14	
NC_004741		Sfle2heaf	HPr/EI/IIA^{Fru}	T1	14	G replaced by A at pos. 3617032);<br>start codon moved to 3615930
NP_837781		Sflexafh	IIA^{Fru}/HPr		14	FPr
NP_836893		Sfleycgc	IIA^{Man}/HPr/EI		16	EI lacks C-t domain
NP_406488	*Yersinia pestis* CO92	Ypestis	EI	T2	10	
NP_406487		Ypestis	HPr	T2	10	
NP_407042		Ypesthn	HPr	R/Ntr	8	NPr
NP_404410		Ypestgaf	GAF/EI	Ntr		
NP_404052		ypescheaf	HPr/EI/IIA^{Fru}	T1	14	
NP_404893		Ypesafh	IIA^{Fru}/HPr		14	FPr

NP_668807	*Yersinia pestis* KIM		Ypestis	EI	T2	10	
NP_668808			Ypestis	HPr	T2	10	
NP_667502			Ypesthn	HPr	R/Ntr	8	NPr
NP_670472			Ypestgaf	GAF/EI	Ntr		
NP_671070			ypeskheaf	HPr/EI/IIA^{Fru}	T1	14	
NP_670187			Ypesafh	IIA^{Fru}/HPr		14	FPr
NP_993935	*Yersinia pestis* biovar		Ypestis	EI	T2	10	
NP_993934	Medievalis str. 91001		Ypestis	HPr	T2	10	
NP_995110			Ypesthn	HPr	R/Ntr	8	NPr
NP_994178			Ypestgaf	GAF/EI	Ntr		
NP_995047			ypesmheaf	HPr/EI/IIA^{Fru}	T1	14	
NP_992660			Ypesafh	IIA^{Fru}/HPr		14	FPr
YP_071226	*Yersinia*		Ypestis	EI	T2	10	
YP_071225	*pseudotuberculosis* IP		Ypestis	HPr	T2	10	
YP_072013	32953		Ypesthn	HPr	R/Ntr	8	NPr
YP_071541			ypseugaf	GAF/EI	Ntr		
YP_069087			ypseheaf	HPr/EI/IIA^{Fru}	T1	14	
YP_069861			Ypesafh	IIA^{Fru}/HPr		14	FPr
NP_820533	*Coxiella burnetii* RSA	*Proteobacteria; Gammaproteobacteria;*	cburngaf	GAF/EI	Ntr		
NP_819769	493	*Legionellales; Coxiellaceae*	cburnet	HPr	R/Ntr	8	NPr
NP_819770			cburnhk	HPrK		8	
YP_096865	*Legionella pneumophila*	*Proteobacteria; Gammaproteobacteria;*	lpnephgaf	GAF/EI	Ntr		
	subsp. *pneumophila* str.	*Legionellales; Legionellaceae*					
YP_094519	Philadelphia 1		Lpneuh	HPr	R/Ntr	8	NPr
YP_128111	*Legionella pneumophila*		lpnelgaf	GAF/EI	Ntr		
YP_125882	str. Lens		Lpneuh	HPr	R/Ntr	8	NPr
YP_125232	*Legionella pneumophila*		lpnepgaf	GAF/EI	Ntr		
YP_122878	str. Paris		Lpneuh	HPr	R/Ntr	8	NPr
NP_872826	*Haemophilus ducreyi*	*Proteobacteria; Gammaproteobacteria;*	hducrey	EI	T2	10	
NP_872827	35000HP	*Pasteurellales; Pasteurellaceae*	hducre	HPr	T2	10	
NP_439854	*Haemophilus influenzae*		hinfluen	EI	T2	10	
NP_439855	Rd KW20		hinfluh	HPr	T2	10	
NP_438609			hinfafhh1/2	IIA^{Fru}/HPr/HPr		14	FPr
YP_088701	*Mannheimia*		msucci	EI	T2	10	
YP_088702	*succiniciproducens*		msuccih	HPr	T2	10	
YP_089372	MBEL55E		msucafhh1/2	IIA^{Fru}/HPr/HPr	FPr	14	
NP_245834	*Pasteurella multocida*		pmulto	EI	T2	10	
NP_245835	subsp. *multocida* str.		pmultoh	HPr	T2	10	
NP_246736	Pm70		pmultafh	IIA^{Fru}/HPr		14	FPr
YP_045212	*Acinetobacter* sp. ADP1	*Proteobacteria; Gammaproteobacteria;*	acinetgaf	GAF/EI	Ntr		
YP_046637		*Pseudomonadales; Moraxellaceae*	Acinaf2he	IIA^{Fru}(2)/HPr/EI	T1	14	
YP_047580			acinhn	HPr	R/Ntr	8	NPr
NP_249028	*Pseudomonas*	*Proteobacteria; Gammaproteobacteria;*	paergaf	GAF/EI	Ntr		
NP_252449	*aeruginosa* PAO1	*Pseudomonadales; Pseudomonadaceae*	paeraghe	IIA^{Glu}/HPr/EI	T1		
NP_252252			Paeraf2he	IIA^{Fru}(2)/HPr/EI	T1	14	
NP_253156			paerhn	HPr	R/Ntr	8	NPr
NP_747246	*Pseudomonas putida*		pputgaf	GAF/EI	Ntr		

NP_742954	KT2440		Pputaf2he	IIA^{Fru}(2)/HPr/EI	T1	14	
NP_743109			pputhn	HPr	R/Ntr	8	NPr
NP_795015	*Pseudomonas syringae*		psyrgaf	GAF/EI	Ntr		
NP_790793	pv. tomato str. DC3000		Psyraf2he	IIA^{Fru}(2)/HPr/EI	T1	14	
NP_794211			psyrhn	HPr	R/Ntr	8	NPr
YP_129077	*Photobacterium*	*Proteobacteria; Gammaproteobacteria; Vibrionales;*	pHProfu	EI	T2	10	
YP_129078	*profundum* SS9	*Vibrionaceae*	pHProf	HPr	T2	10	
YP_131367			pprofhn	HPr	R/Ntr	8	NPr
YP_128803			pHProfgaf	GAF/EI	Ntr		
YP_131890			pHPramhe	IIA^{Man}/HPr/EI	T1	16	
YP_129786			pprofafh	IIA^{Fru}/HPr	FPr	14	
NP_230612	*Vibrio cholerae* O1		vcholer	EI	T2	10	
NP_230613	biovar eltor str. N16961		vcholerh	HPr	T2	10	
NP_232161			vcholhn	HPr	R/Ntr	8	NPr
NP_230321			vcholgaf	GAF/EI	Ntr		
NP_232909			vcholafh	IIA^{Fru}/HPr		14	FPr
YP_205278	*Vibrio fischeri* ES114		vfischer	EI	T2	10	Deleted T at pos. 2136326 in NC_006840 ORF VF1895 and VF1896 in frame. New coding sequence 2135756-2137481
YP_205279							
YP_205277			vfisch	HPr	T2	10	
YP_203766			vfischn	HPr	R/Ntr	8	NPr
YP_203840			vfischgaf	GAF/EI	Ntr		
YP_206900			vfiscafh	IIA^{Fru}/HPr		14	FPr
NP_797173	*Vibrio parahaemolyticus*		vparahaem	EI	T2	10	
NP_797174	RIMD 2210633		vparaha	HPr	T2	10	
NP_796900			vparahgaf	GAF/EI	Ntr		
NP_799053			vparahn	HPr	R/Ntr	8	NPr
NP_800323			vparaafh	IIA^{Fru}/HPr		14	FPr
NP_796745			vparamhe	IIA^{Man}/HPr/EI	T1	16	Start codon moved to 363920 in NC_004603
NP_933770	*Vibrio vulnificus* YJ016		vvulniyj	EI	T2	10	
NP_933771			Vvulnif	HPr	T2	10	
NP_933236			Vvulnhn	HPr	R/Ntr	8	NPr
NP_933469			vvulnygaf	GAF/EI	Ntr		
NP_936762			vvulyafh	IIA^{Fru}/HPr		14	FPr
NP_759221	*Vibrio vulnificus*		vvulnicm	EI	T2	10	
NP_759220	CMCP6		Vvulnif	HPr	T2	10	
NP_759681			Vvulnhn	HPr	R/Ntr	8	NPr
NP_759510			vvulncgaf	GAF/EI	Ntr		Start codon moved to 520015 in NC_004459
NP_762182			vvulcafh	IIA^{Fru}/HPr		14	FPr
NP_298691	*Xylella fastidiosa* 9a5c	*Proteobacteria; Gammaproteobacteria;*	Xyfast9	EI	R	8	
NP_298692		*Xanthomonadales; Xanthomonadaceae*	Xfastid9	HPr	R/Ntr	8	
NP_298695			Xfasthk	HPrK		8	

NP_778853	*Xylella fastidiosa*		xyfasttem	EI	R	8	
NP_778854	Temecula1		xfastidt	HPr	R/Ntr	8	
NP_778857			Xfasthk	HPrK		8	
NP_643288	*Xanthomonas*		xaxono	EI	R	8	
NP_643287	*axonopodis* pv. *citri* str.		Xaxonoh	HPr	R/Ntr	8	
NP_642816	306		xaxoafhe	IIA^{Fru}/HPr/EI	T1	14	
NP_643284			xaxonhk	HPrK		8	
NP_638157	*Xanthomonas*		xcamp	EI	R	8	
NP_638156	*campestris* pv.		xcampes	HPr	R/Ntr	8	
NP_637724	*campestris* str. ATCC		xcamafhe	IIA^{Fru}/HPr/EI	T1	14	
NP_638153	33913		xcamphk	HPrK		8	
YP_199916	*Xanthomonas oryzae* pv. *oryzae* KACC10331		xoryz	EI	R	8	Corrected start codon to 1312175 in NC_006834
YP_199917			Xaxonoh	HPr	R/Ntr	8	
YP_201449			xoryafhe	IIA^{Fru}/HPr/EI	T1	14	
YP_199920			xoryzhk	HPrK		8	
NP_602593	*Fusobacterium*	*Fusobacteria; Fusobacteria* (class); *Fusobacterales;*	fnucleat	EI	T2	10	
NP_602594	*nucleatum* subsp.	*Fusobacteriaceae*	Fnuclh1	HPr	T2	10	
NP_602582	*nucleatum* ATCC 25586		Fnuclh2	HPr			
NP_603909			Fnuclhk1/2	HPrK/HPrK		12	
YP_077104	*Symbiobacterium*	*Actinobacteria; Actinobacteria* (class); unclassified	sythermoph	EI	T1	13	
YP_077105	*thermophilum* IAM	*Actinobacteria*	Syther2	HPr		13	
YP_074784	14863		Syther1	HPr		13	
NP_939780	*Corynebacterium*	*Actinobacteria; Actinobacteria* (class);	cdiphter	EI	T1	13	
NP_939783	*diphtheriae* NCTC	*Actinobacteridae; Actinomycetales;*	cdiphth	HPr		13	
NP_940637	13129	*Corynebacterineae; Corynebacteriaceae*	cdiphtamh	IIA^{Man}/HPr		13	
NP_738436	*Corynebacterium*		ceffici	EI	T1	13	
NP_738440	*efficiens* YS-314		ceffih	HPr		13	
YP_226175	*Corynebacterium glutamicum* ATCC		cglutam	EI	T1	13	
YP_226179	13032		cgluth	HPr		13	
YP_119068	*Nocardia farcinica* IFM 10152	*Actinobacteria; Actinobacteria* (class); *Actinobacteridae; Actinomycetales;*	nfarcini	EI	T1	13	
YP_119064		*Corynebacterineae; Nocardiaceae*	nfarcinh	HPr		13	
YP_061276	*Leifsonia xyli* subsp. *xyli* str. CTCB07	*Actinobacteria; Actinobacteria* (class); *Actinobacteridae; Actinomycetales; Micrococcineae;*	lxyli	EI	T1	13	Start codon at 80598 in NC_006087
YP_061277		*Microbacteriaceae*	lxylih	HPr		13	
YP_061966			lxylamh	IIA^{Man}/HPr		13	
YP_055064	*Propionibacterium*	*Actinobacteria; Actinobacteria* (class);	pacnes	EI	T1	13	
YP_055065	*acnes* KPA171202	*Actinobacteridae; Actinomycetales;*	Pacneh1	HPr		13	
YP_054857		*Propionibacterineae; Propionibacteriaceae*	Pacneh2	HPr		13	
NP_828150	*Streptomyces avermitilis* MA-4680	*Actinobacteria; Actinobacteria* (class); *Actinobacteridae; Actinomycetales; Streptomycineae;*	savermit	EI	T1	13	
NP_823594		*Streptomycetaceae*	savermh	HPr		13	
NP_625674	*Streptomyces coelicolor*		scoelic	EI	T1	13	
NP_629964	A3(2)		scoelih	HPr		13	
NP_695612	*Bifidobacterium longum*	*Actinobacteria; Actinobacteria* (class);	blongum	EI	T1	13	

NP_695613	NCC2705	*Actinobacteridae; Bifidobacteriales; Bifidobacteriaceae*	blong	HPr		13	
NP_868449	*Rhodopirellula baltica* SH 1	*Planctomycetes; Planctomycetacia; Planctomycetales; Planctomycetaceae*	rbaltic	EI	R	9	
NP_865264			Rbaltic2	EI	R	9	
NP_868447			rbaltich	HPr	R/Ntr	9	
NP_219843	*Chlamydia trachomatis* D/UW-3/CX	*Chlamydiae/Verrucomicrobia* group; *Chlamydiae; Chlamydiae* (class); *Chlamydiales; Chlamydiaceae*	chtrachom	EI	R	9	
NP_219844			chtrach	HPr	R/Ntr	9	
NP_296989	*Chlamydia muridarum* Nigg		cmurida	EI	R	9	
NP_296990			cmuridh	HPr	R/Ntr	9	
NP_829199	*Chlamydophila caviae* GPIC		chcavia	EI	R	9	
NP_829198			chcaviah	HPr	R/Ntr	9	
NP_224246	*Chlamydophila pneumoniae* CWL029		chpneuc	EI	R	9	
NP_224245			Chpneuh	HPr	R/Ntr	9	
NP_300099	*Chlamydophila pneumoniae* J138		chpneuj	EI	R	9	
NP_300098			Chpneuh	HPr	R/Ntr	9	
NP_876318	*Chlamydophila pneumoniae* TW-183		chpneut	EI	R	9	
NP_445280			Chpneuh	HPr	R/Ntr	9	
YP_007475	*Candidatus Protochlamydia amoebophila UWE25*	*Chlamydiae/Verrucomicrobia* group; *Chlamydiae*; *Chlamydiae* (class); *Chlamydiales; Parachlamydiaceae*	parachla	EI	R	9	
YP_007474			Parach1	HPr	R/Ntr	9	
YP_008176			Parach2	HPr	R/Ntr	9	
YP_007473			parachk	HPrK		9	
YP_001590	*Leptospira interrogans* serovar Copenhageni str. Fiocruz L1-130	*Spirochaetes; Spirochaetes* (class); *Spirochaetales; Leptospiraceae*	linterfio	EI	R	9	
YP_001506			lintch	HPr		9	Start codon corrected to 1909612 in NC_005823
YP_001505			Lintehk	HPrK		9	
NP_712488	*Leptospira interrogans* serovar Lai str. 56601		linterlai	EI	R	9	
NP_712583			lintlh	HPr		9	
NP_712584			Lintehk	HPrK		9	
NP_212692	*Borrelia burgdorferi* B31	*Spirochaetes; Spirochaetes* (class); *Spirochaetales; Spirochaetaceae*	bburgdo	EI	T2	9/10	
NP_212691			Bburgd[18]	HPr	T2	9/10	
NP_212582			Bburgdh2	HPr		9	
YP_072997	*Borrelia garinii* PBi		bgarini	EI	T2	9/10	
YP_072996			Bburgd	HPr	T2	9/10	
YP_072891			Bgarinh2	HPr		9	
NP_970701	*Treponema denticola* ATCC 35405		tdentic	EI		9	
NP_971901			tdentih	HPr		9	
NC_002967			tdenthk	HPrK		9	Inserted N after pos. 461, A after pos. 476, and G after pos. 568 in NC_002967 REGION: complement (1332424-1333377)
B71379	*Treponema pallidum* subsp. *pallidum* str. Nichols		tpallid	EI (pos. mut. En NC_000919)		9	Deleted TA at pos. 624327-624328 in NC_000919; coding sequence 623682-625738
NP_219027			tpallih	HPr		9	

NP_219029			tpallhk	HPrK		9	
NP_661110	*Chlorobium tepidum*	*Bacteroidetes/Chlorobi* group; *Chlorobi; Chlorobia*;	ctepidum	EI	R	9	
NP_663085	TLS	*Chlorobiales; Chlorobiaceae;*	ctepid	HPr	R/Ntr	9	
NP_662516		*Chlorobium/Pelodictyon* group	ctepihk	HPrK		9	
NP_284935	*Deinococcus radiodurans* R1	*Deinococcus-Thermus; Deinococci; Deinococcales; Deinococcaceae*	dradafhe	IIA^{Fru}/HPr/EI	T1	14	C inserted after pos. 94331; start codon moved to pos. 94577 in NC_000958; located in plasmid MP1
YP_134786	*Haloarcula marismortui*	*Archaea; Euryarchaeota; Halobacteria;*	hmaris	EI	T1	14	Plasmid pNG700
YP_134785	ATCC 43049	*Halobacteriales; Halobacteriaceae*	hmarish	HPr		14	Plasmid pNG700

[a] Species lacking of EI, HPr or HPrK/P homologues are not indicated
[b] Identical sequences share the same alias. Only one representative has been utilised in this study.
[c] Groups identified in the phylogenetic analyses (see Figs. 2, 3 and 4 and Suppl. Figs. 5, 6 and 7). Sequences in uncertain positions are left in blank.
[d] Numbers indicate Supplemental Figures where the corresponding clusters are depicted.
[e] *Bacillus anthracis* str. Ames, *Bacillus anthracis* str. Sterne and *Bacillus anthracis* str. A2012 encode identical EI, HPr and HPrK/P proteins.
[f] *Listeria monocytogenes* str. 4b F2365 encode identical EI, HPr and HPrK/P proteins
[g] *Staphylococcus epidermidis* RP62A encode identical EI, HPr and HPrK/P proteins
[h] *Streptococcus pneumoniae* TIGR4 encode identical EI, HPr and HPrK/P proteins
[i] *Streptococcus pyogenes* MGAS10394 and *Streptococcus pyogenes* M1 GAS encode identical EI, HPr and HPrK/P proteins
