# Supplementary material for: Unraveling the evolutionary history of the phosphoryl-transfer chain of the phosphoenolpyruvate:phosphotransferase system through phylogenetic analyses and genome context
Source: BMC Evol Biol. 2008 May 16;8:147. doi: 10.1186/1471-2148-8-147 (PMC2405797; doi:10.1186/1471-2148-8-147)
Supplement: Additional file 2 — Supplementary figures. Figure S1: likelihood mapping analysis. Figures S2 and S3: maximum likelihood phylogenetic trees for EI and HPr sequences, respectively. Figure S4: schematic representation of the proposed ancestral rpoN gene cluster of Proteobacteria. Figure S5: comparison of the topologies of phylogenetic trees for 16S and EI sequences belonging to groups R, Ntr and T (VPES). Figure S6: phylogenetic reconstruction of 16S rRNA sequences of the strains used in this study harbouring genes encoding EI, HPr or HPrK. Figure S7: ptsH or ptsI gene clusters of Actinobacteria. Figure S8: gene clusters containing FPr encoding genes and related homologues. Figure S9: sequence alignment of the IIAFru and the intervening domain of FPr proteins, Acinetobacter sp. FruB protein, and the tandem IIAFru domains of Pseudomonas FruA proteins. Figure S10: ptsK gene clusters of Bacillales. Figure S11: ptsK gene clusters of Firmicutes and F. nucleatum. Figure S12: ptsH or ptsI gene clusters of Firmicutes, VPES, Borrelia and F. nucleatum. [file 1471-2148-8-147-S2.pdf]

Figure S1. Likelihood mapping analysis. The regions at the corners of the triangles correspond to the three possible tree topologies for a quartet; the lateral regions to partly resolved trees and the central region to unresolved trees. The numbers indicate the percentage of quartets falling in each region.

Figure S2. Maximum likelihood phylogenetic tree for EI sequences used in this study. The tree is arbitrarily rooted with the group T2. The main groups derived from the analyses are indicated. Support values for the bootstrap analysis by maximum likelihood and for bayesian "*a posteriori*" probabilities are given for those nodes with at least 50% of bootstrap support or 100% "*a posteriori*" probability. # indicates nodes with 100% bootstrap and 100% "*a posteriori*" probability.

Figure S3. Maximum likelihood topology of the HPr sequences used in this study. Support of nodes is indicated as in Figure S2.

Figure S4. Schematic representation of the proposed ancestral *rpoN* gene cluster of *Proteobacteria*. Representative clusters of the major divisions of *Proteobacteria* are depicted. Colors indicate homologous genes or domains.

Figure S5. Panel A: Comparison of phylogenetic trees for 16S rRNA and EI of species harbouring EI^{R}. The trees have been arbitrarily rooted with the corresponding sequences of *Rhodopirellula baltica*.. Panel B: Comparison of phylogenetic trees for 16S rRNA and EI of species harbouring EI^{Ntr}. The trees have been arbitrarily rooted with the corresponding sequences of *Geobacter sulfurreducens*. Panel C: Comparison of phylogenetic trees for 16S rRNA and EI^{T} of species of the VPES group (excepting *Shewanella oneidensis*). The trees have been arbitrarily rooted with the corresponding *Pasteurellaceae* sequences.

Figure S6. Maximum likelihood phylogenetic tree for 16S rRNA sequences of the species used in this study. Support values for the bootstrap analysis by maximum likelihood are given for those nodes with at least 60% of support. The length of the *Haloarcula marismortui* branch has been shortened.

Figure S7. Gene clusters containing *ptsH* or *ptsI* homologues present in *Actinobacteria*. Colors indicate homologous genes or domains. Dashed lines indicate putative pseudogenes.

Figure S8. Panel A; Clusters containing genes encoding FPr and related fructose class PTS proteins. Panel B; Clusters containing genes encoding homologues of the *E. coli* Frw PTS. Colors indicate homologous genes or domains. Dashed rectangles indicate homologous domains possibly non functional.

Figure S9. Sequence alignment of the IIA^{Fru} and the intervening domain of FPr proteins, *Acinetobacter* sp. *FruB* protein, and the tandem IIA^{Fru} domains of *Pseudomonas FruA* proteins. Phosphorylatable His residues are indicated in red.

Figure S10. Gene clusters containing *ptsK*, *crh* or other *ptsH* paralogues present in *Bacillales*. Colors indicate homologous genes or domains.

Figure S11. Gene clusters containing *ptsK* present in *Clostridia*, *Lactobacillales*, *Mollicutes* and *Fusobacterium nucleatum*. Colors indicate homologous genes or domains.

Figure S12. Gene clusters containing *ptsH* or *ptsI* (EI^{T}) homologues present in *Borrelia*, *Firmicutes*, *F. nucleatum* and VPES. Colors indicate homologous genes or domains.

Supplementary Fig. S1

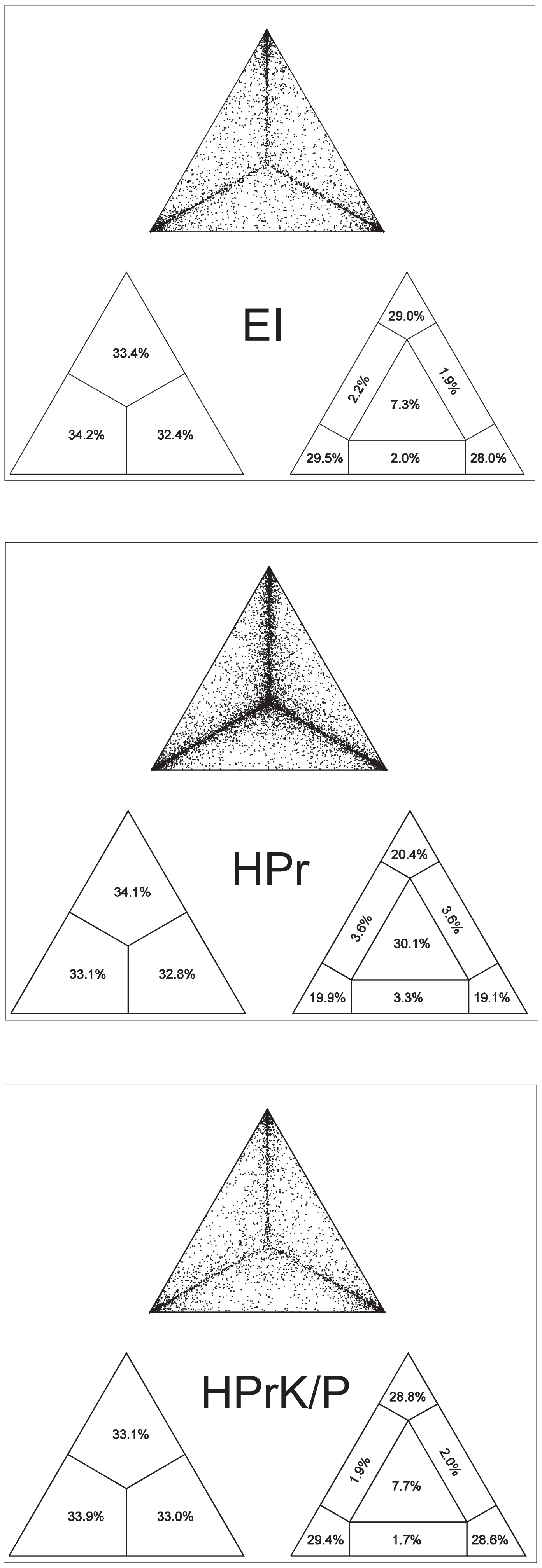

Comas et al.

Supplementary Fig. S2

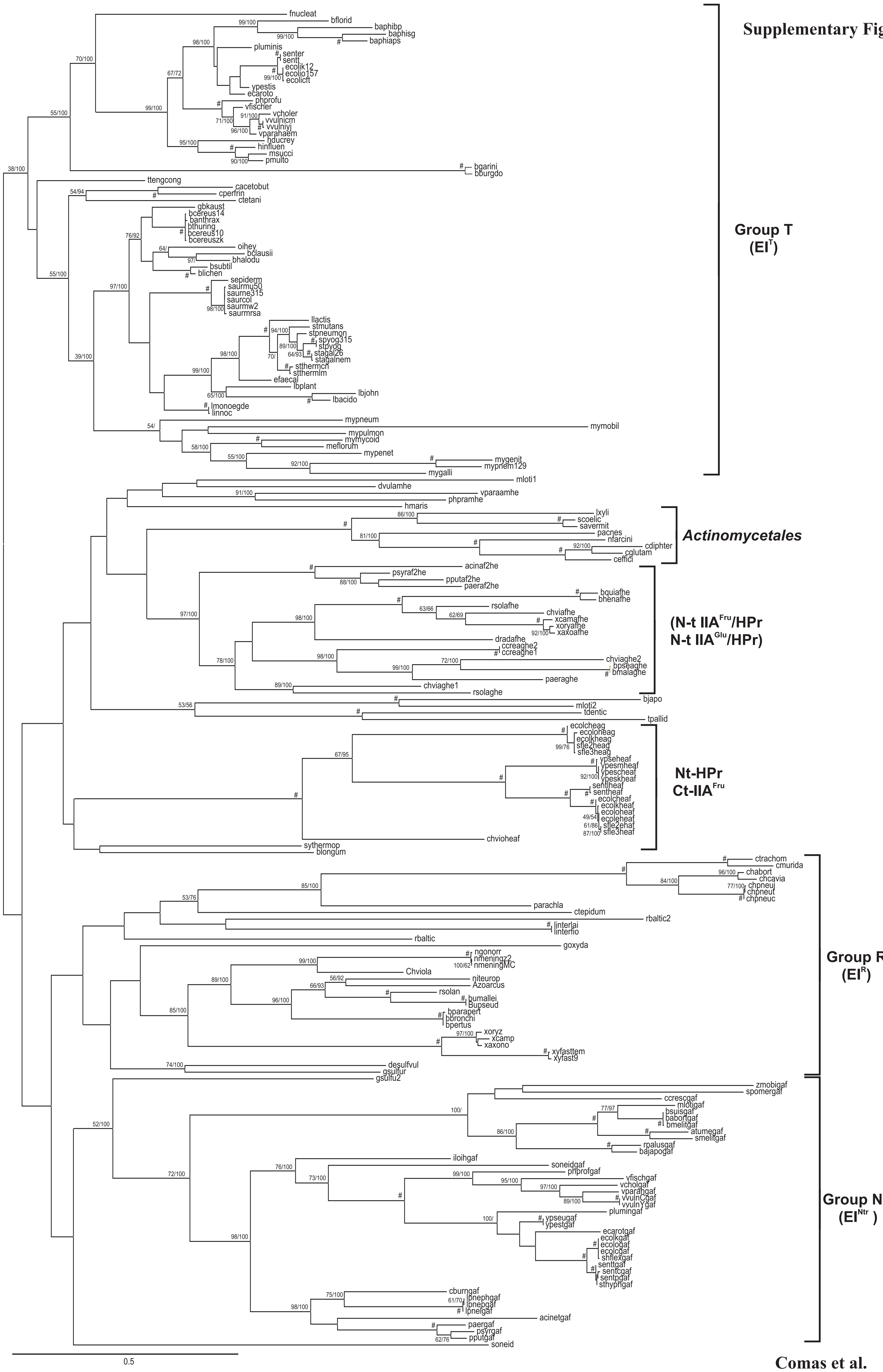

Comas et al.

Supplementary Fig. S3

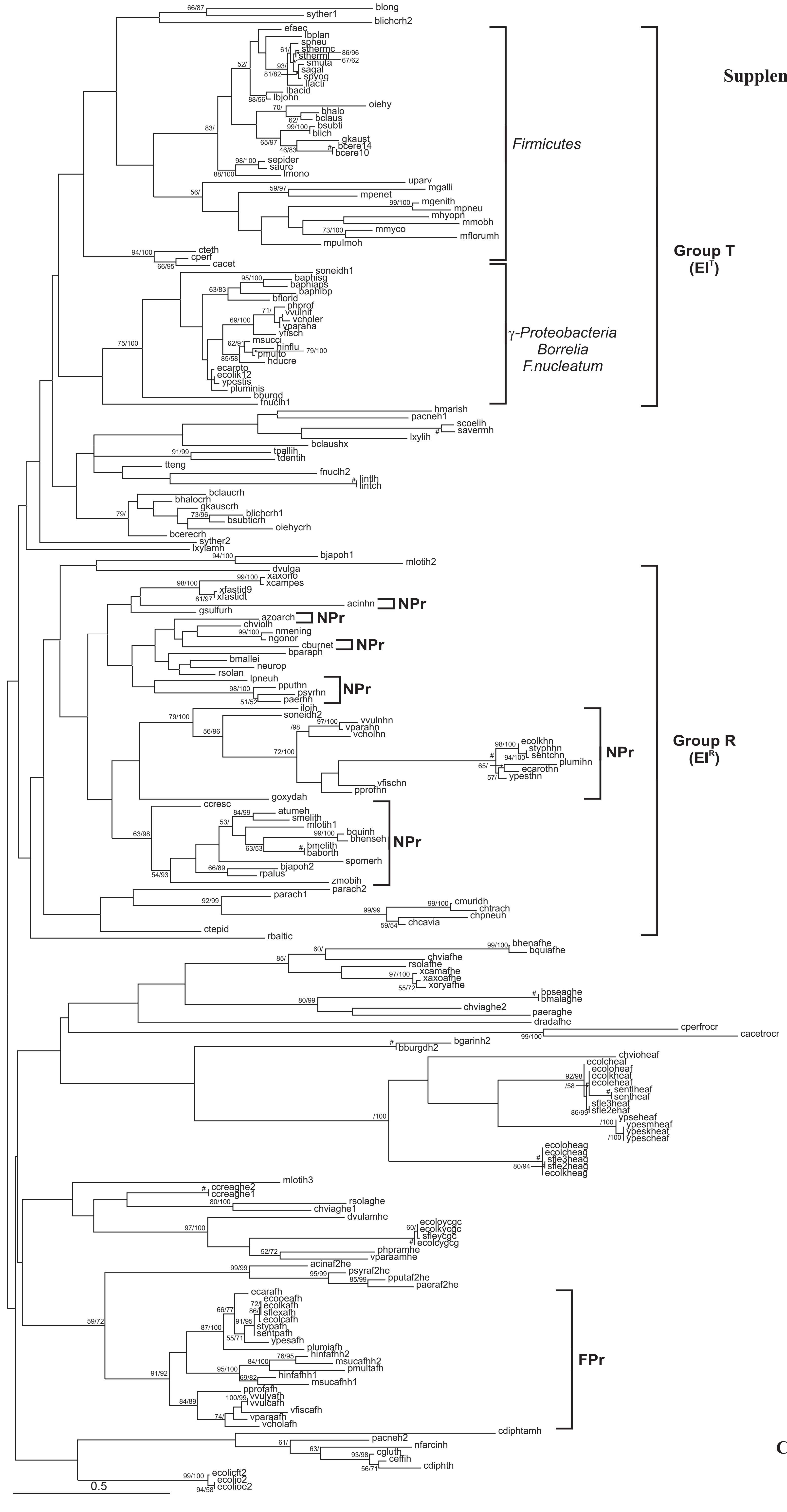

Comas et al.

Supplementary Fig. S4

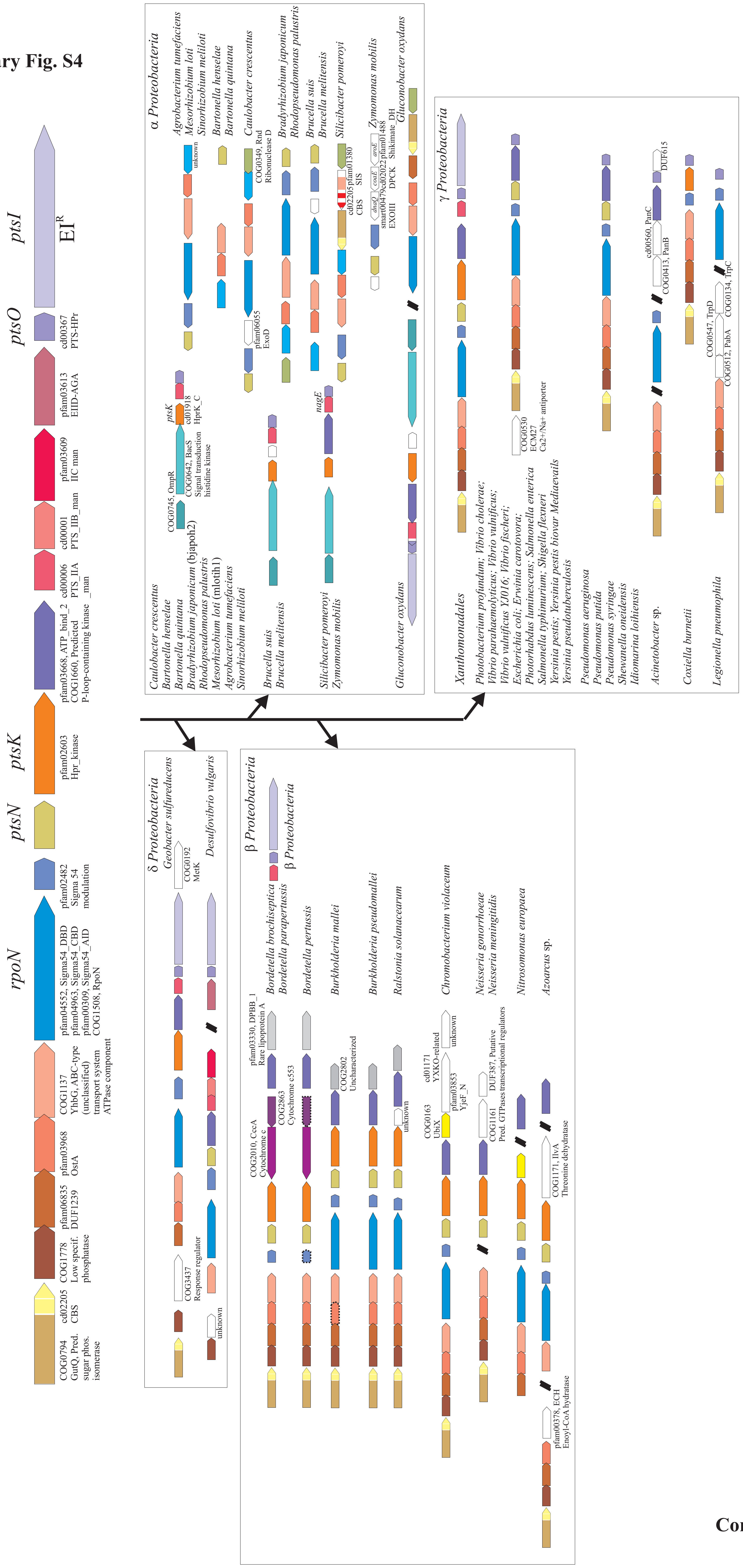

Comas et al.

Supplementary Fig. S5

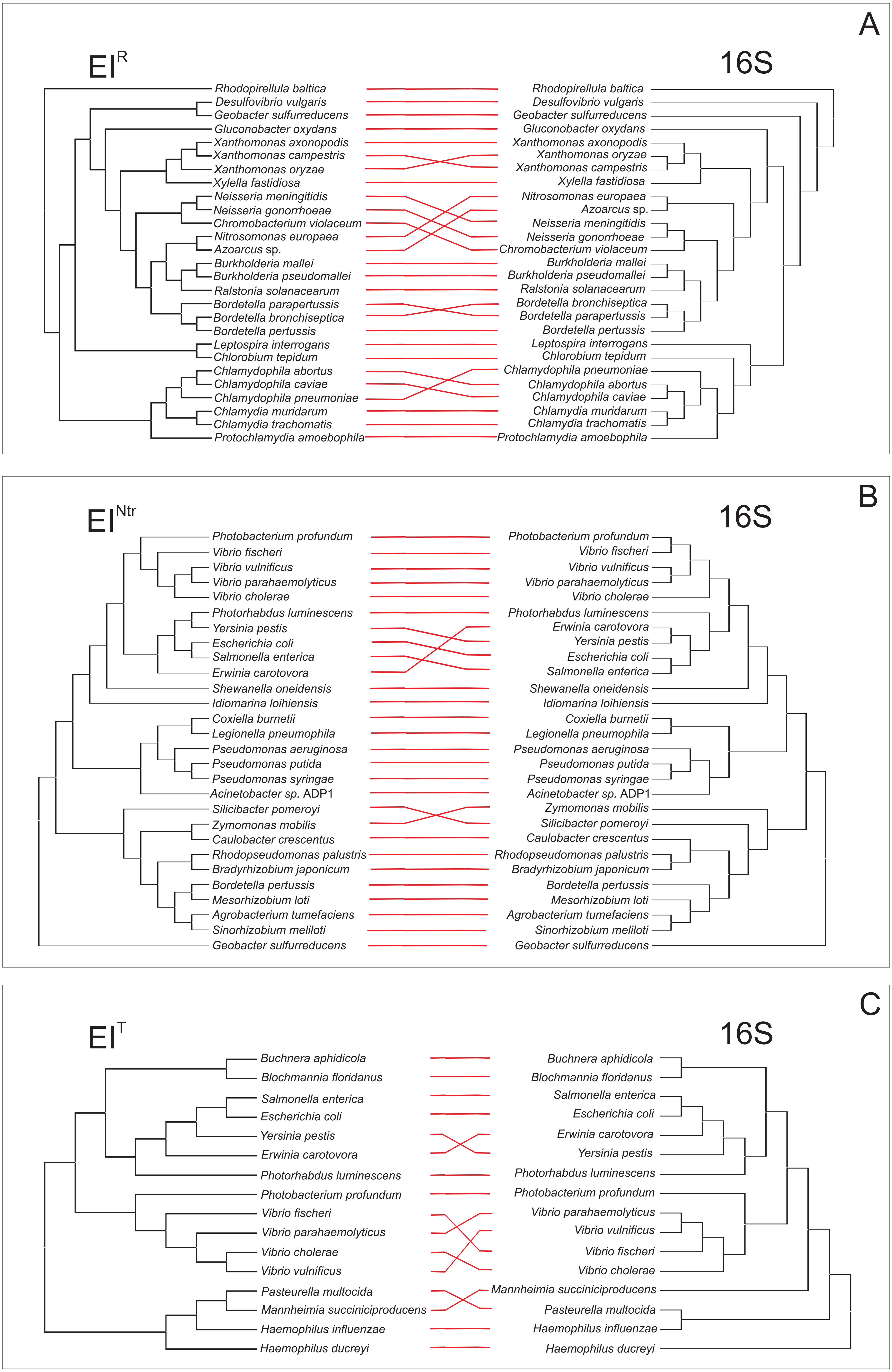

Comas et al.

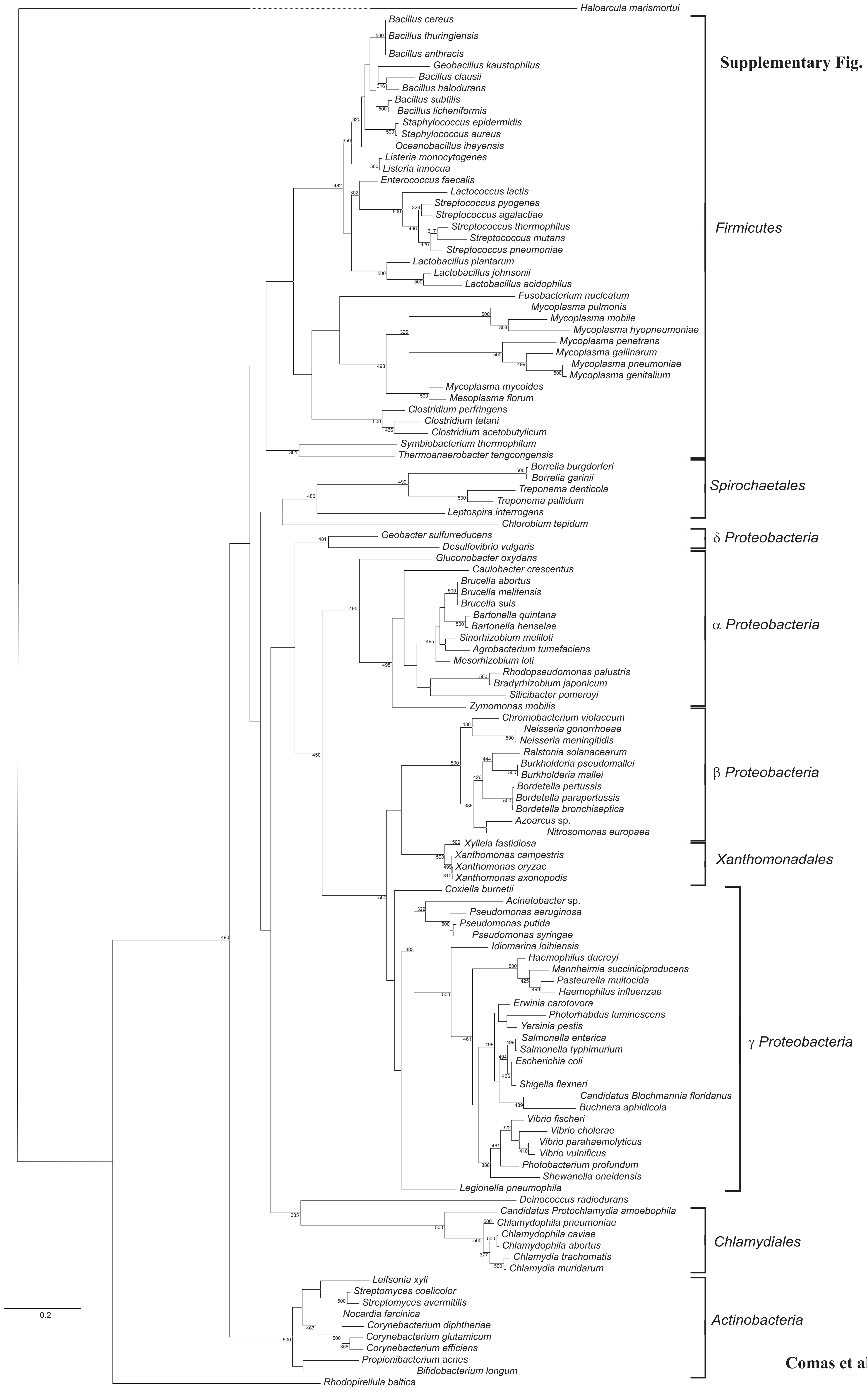

Supplementary Fig. S6

Comas et al.

Supplementary Fig. S7

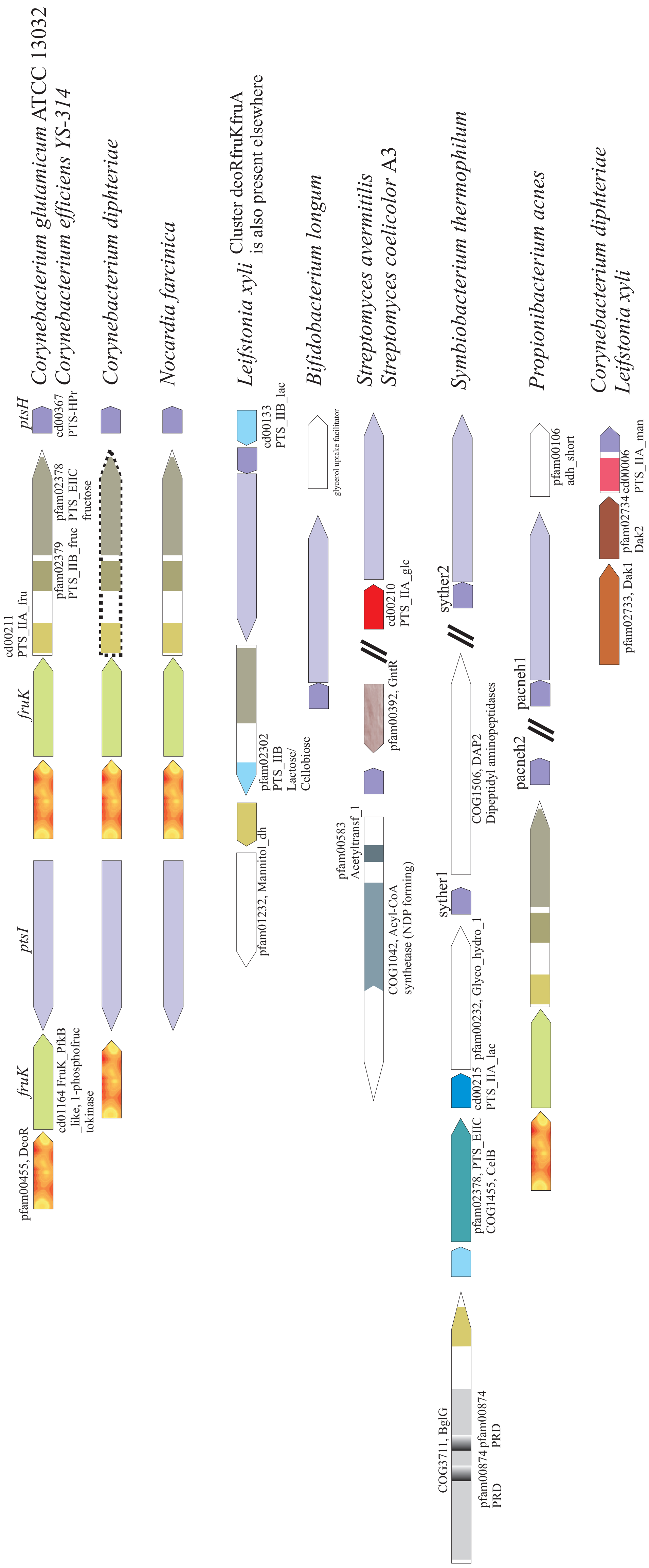

Comas et al.

Supplementary Fig. S8

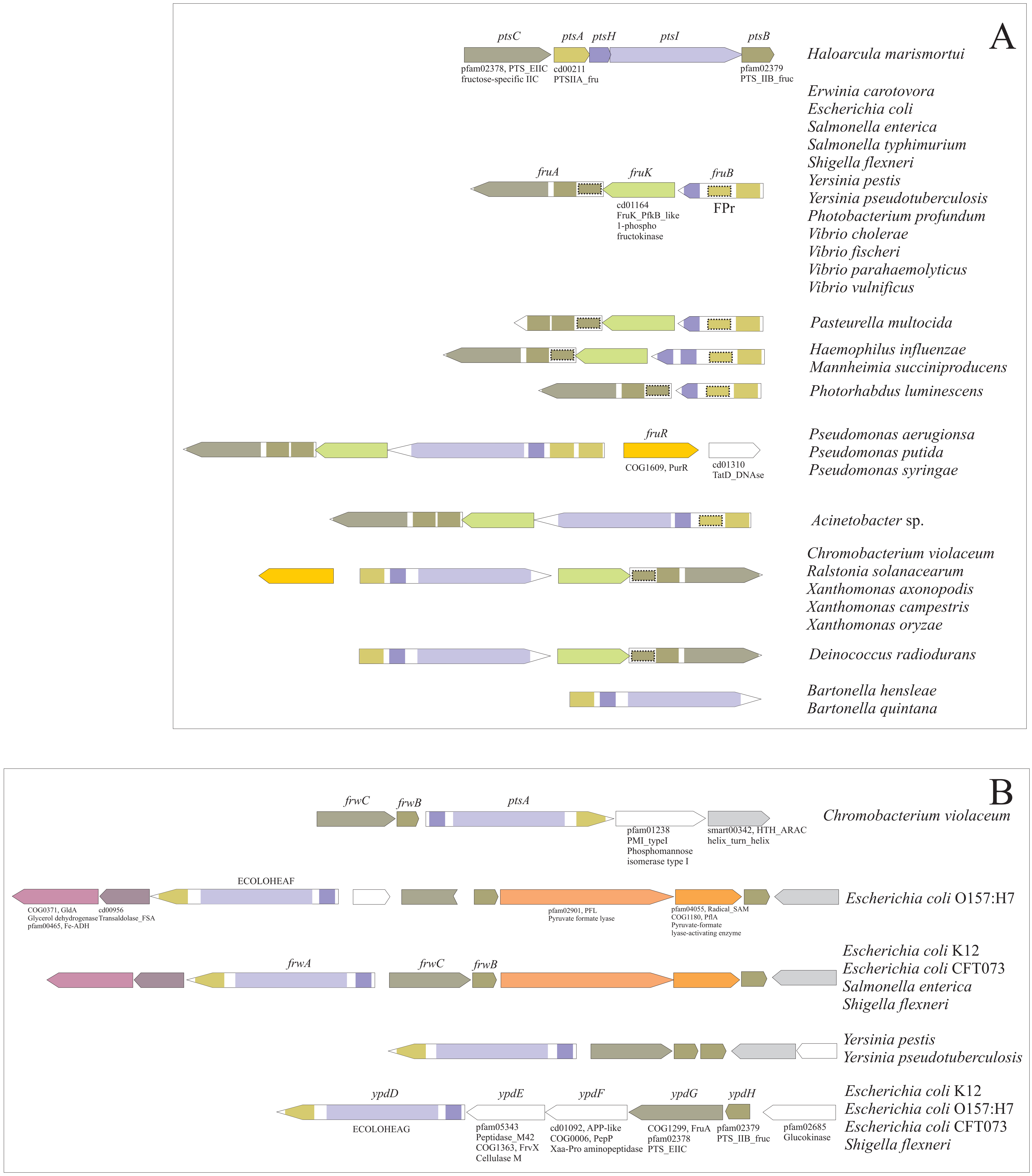

Comas et al.

Supplementary Fig. S9

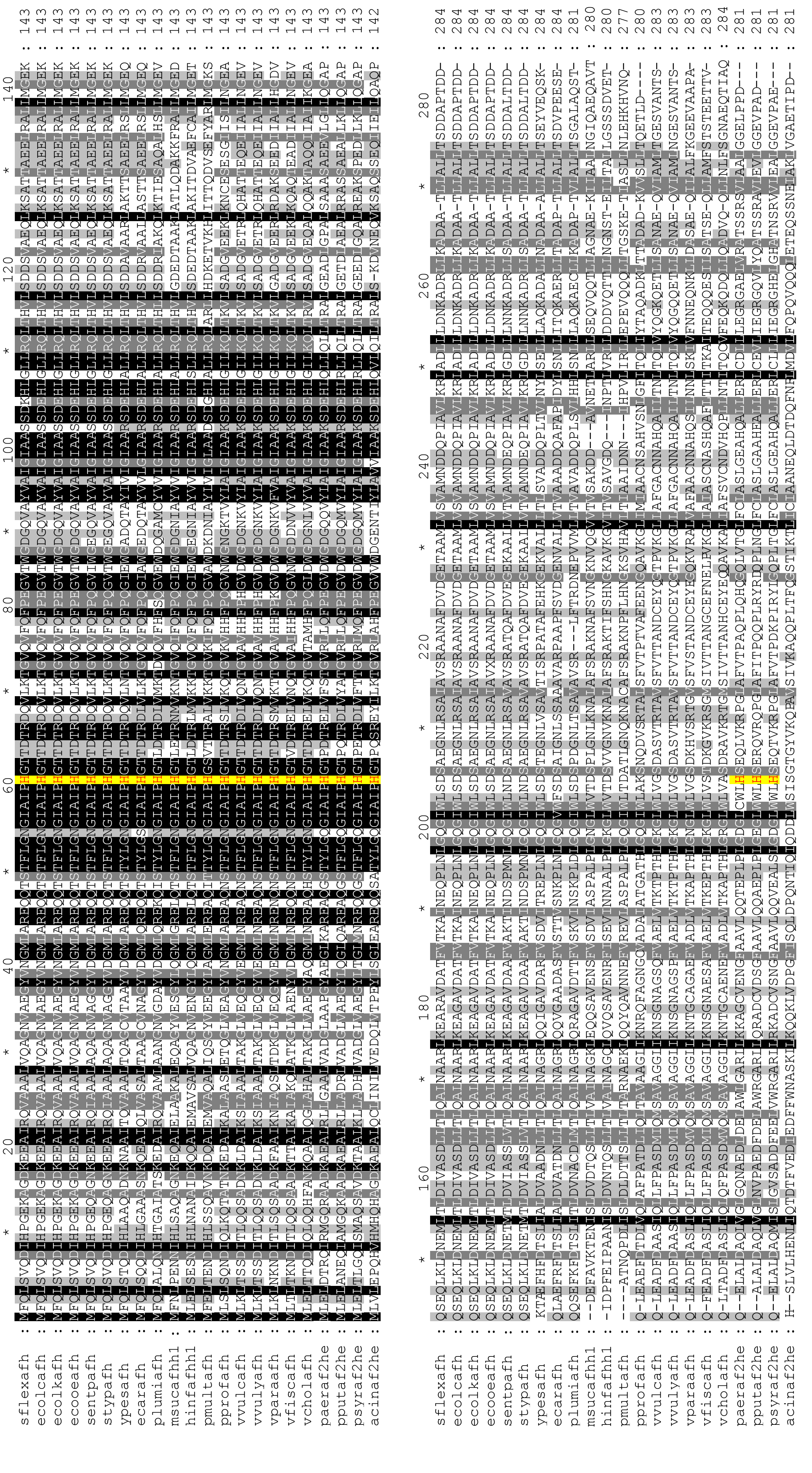

Comas et al.

Supplementary Fig. S10

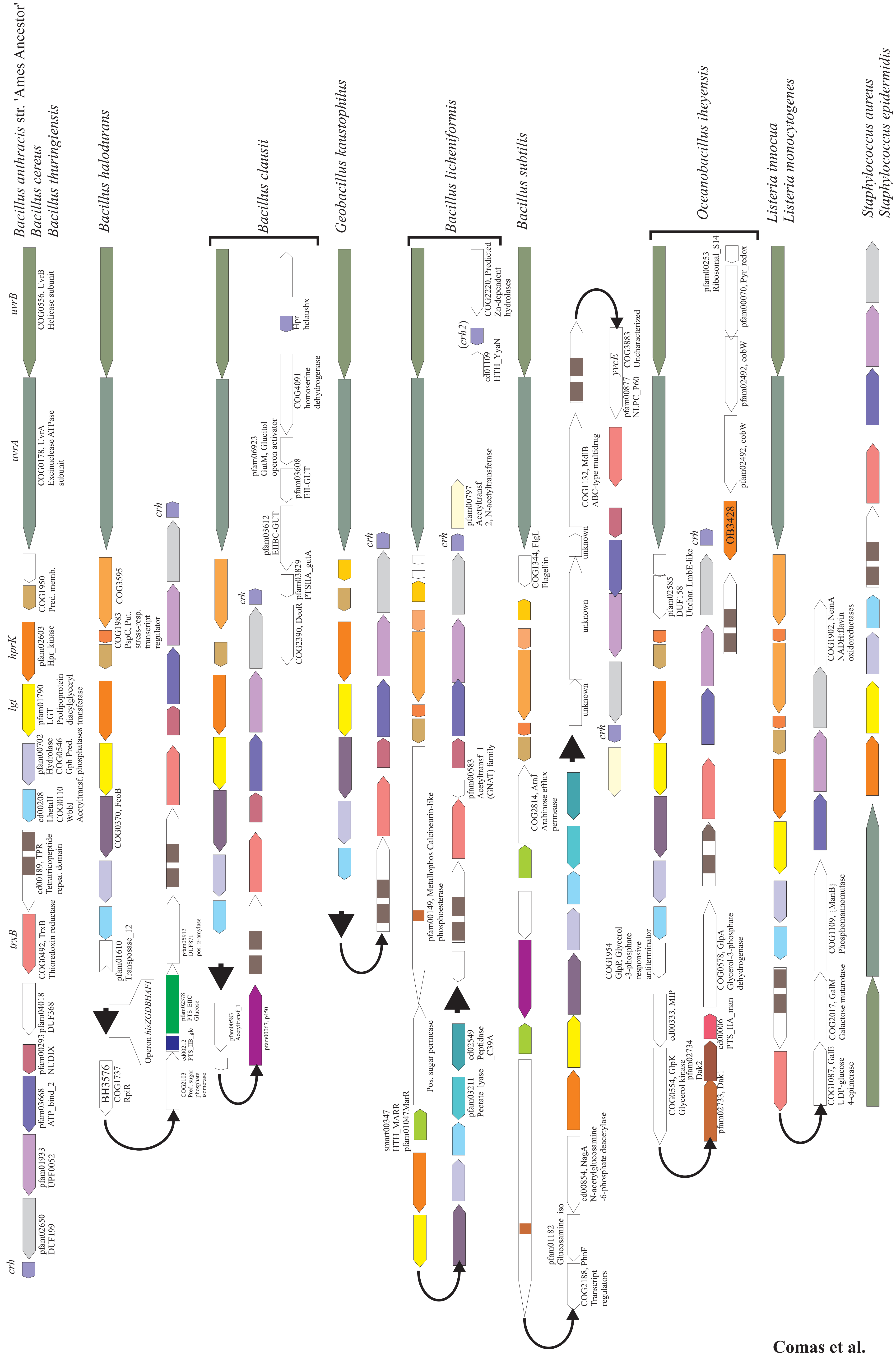

Comas et al.

Supplementary Fig. S11

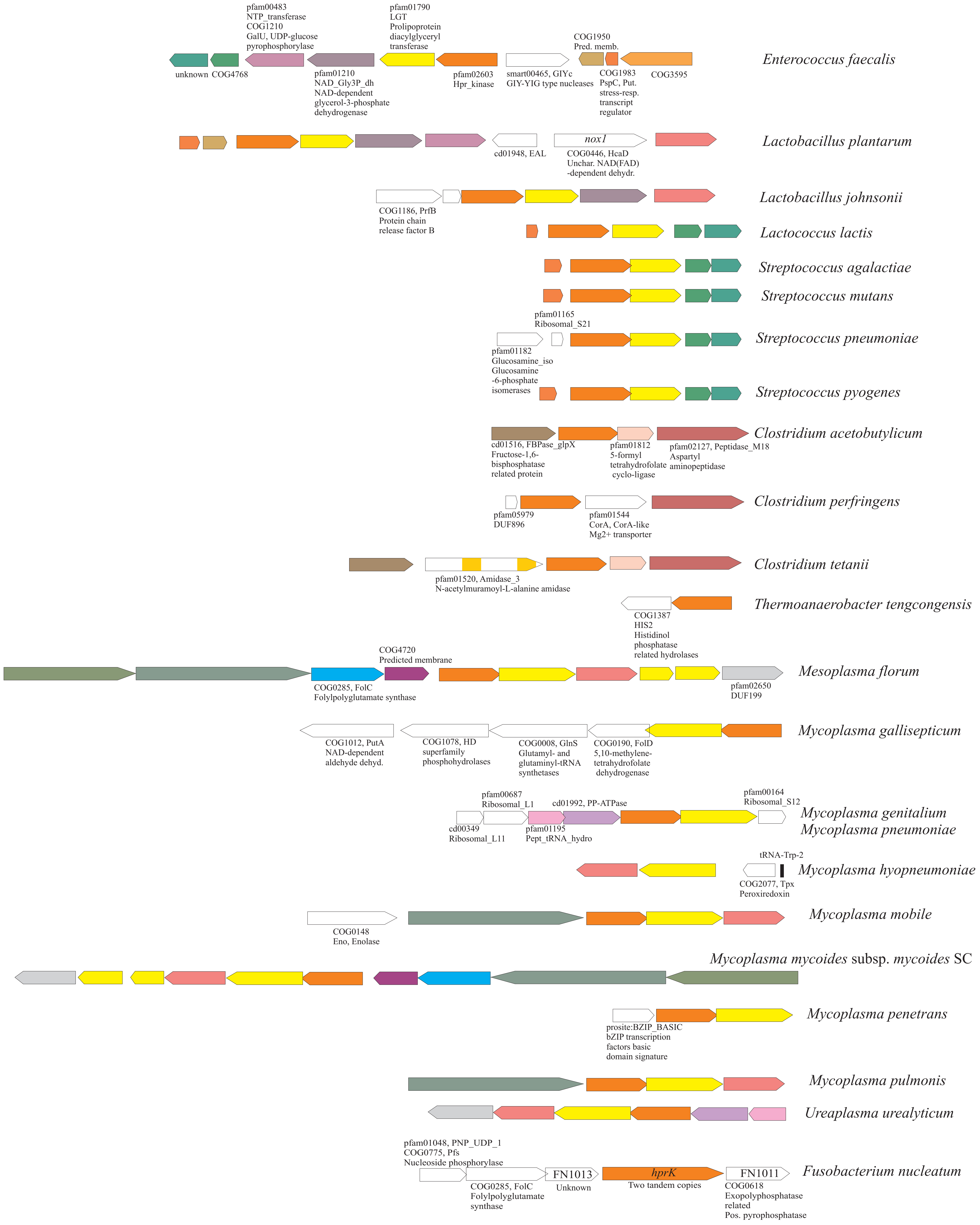

Comas et al.

Supplementary Fig. S12

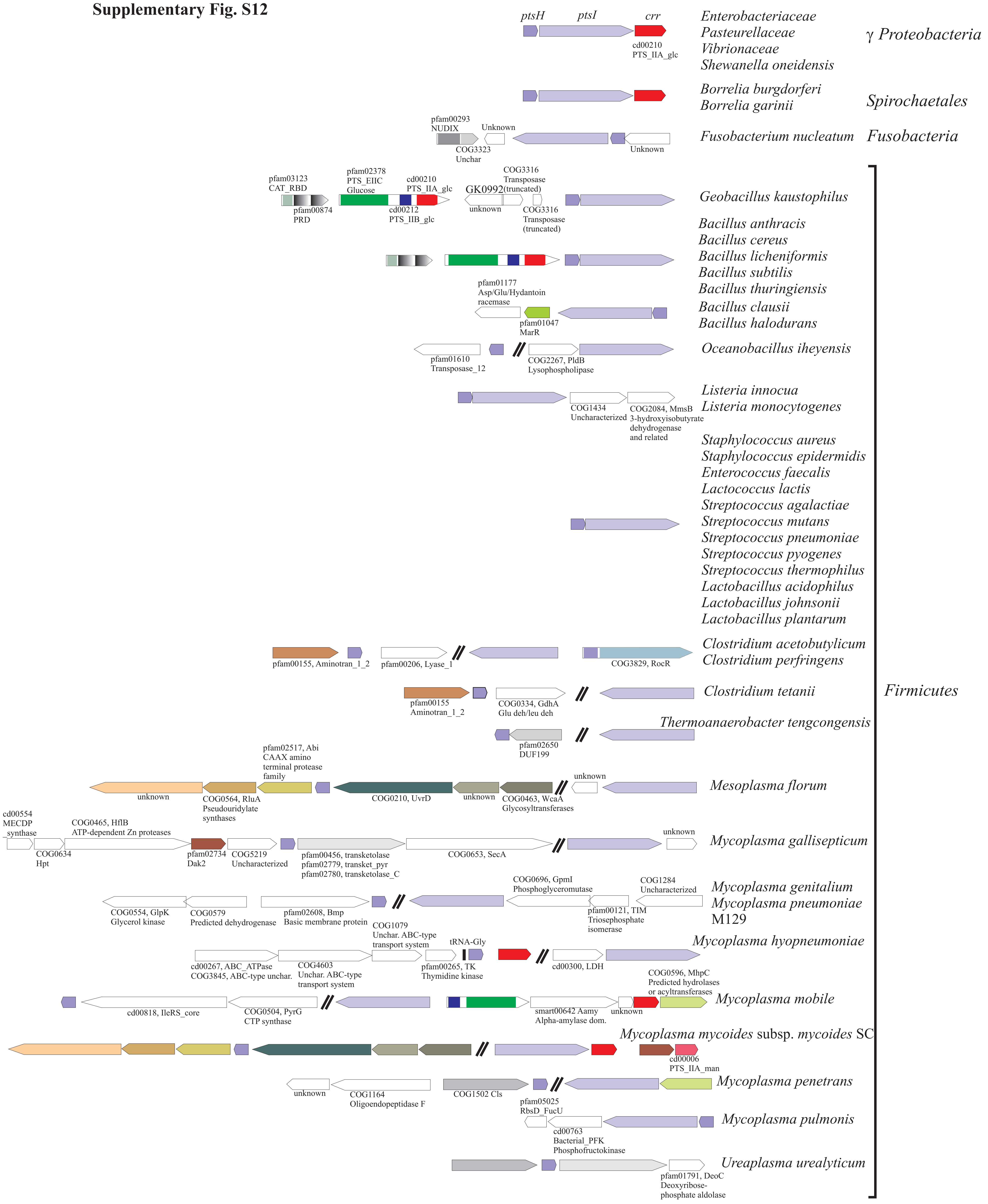

Comas et al.
